# Prescribing trends of glaucoma medication in Korea from 2007 to 2020: A nationwide population-based study

**DOI:** 10.1371/journal.pone.0305619

**Published:** 2024-07-11

**Authors:** Kyeong Ik Na, Won June Lee, Youn Joo Choi, Sung Pyo Park

**Affiliations:** 1 Department of Ophthalmology, Kangdong Sacred Heart Hospital, Hallym University College of Medicine, Seoul, Korea; 2 Department of Ophthalmology, Hanyang University Seoul Hospital, Hanyang University College of Medicine, Seoul, Korea; Alexandria University Faculty of Medicine, EGYPT

## Abstract

**Purpose:**

Investigating long-term trends in glaucoma medication.

**Methods:**

All patients diagnosed with glaucoma and prescribed glaucoma eye drops between 2007 and 2020 in Korea’s Health Insurance Review and Assessment Service database participated in this study. A weight was assigned to each prescription using the reciprocal of the total number of prescriptions received by the individual in that year. The number of patients who received each type of glaucoma eye drop prescription was calculated by summing the weights for each year.

**Results:**

During the study period, prostaglandin analog eye drop monotherapy was the most frequently given type of glaucoma eye drop prescription. Until 2008, the second most frequently given type of glaucoma eye drop prescription was beta blocker eye drop monotherapy; thereafter, it changed to carbonic anhydrase inhibitor/beta blocker fixed-combination eye drop monotherapy. The prescription proportion of single-ingredient glaucoma eye drops decreased (-1.290%/year, *P* < 0.001), whereas that of fixed-combination glaucoma eye drops increased (1.291%/year, *P* < 0.001). The number of glaucoma eye drops prescribed per patient remained constant (-0.00030/year, *P* = 0.167) with an average of 1.302, while the number of active ingredients prescribed per patient increased (0.01737/year, *P* < 0.001) from 1.659 in 2007 to 1.896 in 2020.

**Conclusion:**

Over 14 years, there was no change in the number of glaucoma eye drops prescribed to individual patients in Korea. However, the number of active ingredients prescribed increased owing to the increased prescription of fixed-combination eye drops. The current trends in glaucoma medication are expected to help establish future treatment strategies.

## Introduction

Glaucoma is a progressive optic neuropathy characterized by loss of ganglion cells and subsequent visual field defects [1]. Intraocular pressure is considered a very important risk factor for the development and progression of glaucoma [2–4]. Therefore, most glaucoma treatments focus on lowering intraocular pressure to preserve the remaining ganglion cells and their axons [5].

In Korea, glaucoma eye drops with the following five active ingredients are used: alpha agonists (apraclonidine and brimonidine), beta blockers (betaxolol, carteolol, and timolol), carbonic anhydrase inhibitors (brinzolamide and dorzolamide), parasympathomimetics (pilocarpine), and prostaglandin analogs (bimatoprost, latanoprost, tafluprost, and travoprost). Glaucoma eye drops containing latanoprostene and omidenepag isopropyl were introduced in 2022. Ophthalmologists usually prescribe glaucoma eye drops that have only one of these active ingredients. However, when further intraocular pressure-lowering effects are needed, they prescribe several glaucoma eye drops with different active ingredients. Fixed-combination glaucoma eye drops (combining two active ingredients into one eye drop) are also used.

Investigating the prescription of glaucoma eye drops can provide insights into the factors physicians consider when prescribing glaucoma eye drops to patients. Additionally, analyzing long-term changes in prescription patterns aids in establishing future glaucoma treatment strategies.

Although several studies have examined trends in glaucoma medication, most were limited to certain patient groups and focused only on the number of glaucoma eye drops prescribed [6–9]. However, since individual patients use glaucoma eye drops, it is important to analyze glaucoma eye drop prescription in each individual patient, whether two or more medications were prescribed, and which combinations were prescribed more frequently in each situation.

Therefore, this study investigated glaucoma eye drop prescription for patients with glaucoma using the Health Insurance Review and Assessment Service (HIRA) database, which includes all medical prescription data for the entire Korean population. Fourteen years of HIRA data were analyzed to evaluate trends in glaucoma medication use in Korea.

## Methods

This study was approved by the Institutional Review Board of Kangdong Sacred Heart Hospital and complied with the tenets of the Declaration of Helsinki [10]. Informed consent was not required.

### Data source

The entire Korean population (approximately 53 million people) is covered by compulsory health insurance. Overall, 97.1% of the total population are enrolled in the National Health Insurance Service, and the remaining 2.9% are enrolled in the Medical Aid Program and health care benefits for veterans. All medical information, including diagnosis, procedures, and prescription data, is stored in the HIRA database.

We searched the HIRA database to investigate glaucoma eye drops prescribed to Korean patients. We accessed this data between August 1, 2022 and July 31, 2023.

### Participants

We extracted and analyzed the data of all patients diagnosed with glaucoma and prescribed glaucoma eye drops in the HIRA database between 2007 and 2020.

In the database, diagnoses are coded using the Korean Standard Classification of Disease (KCD), version 5 (modified from the International Statistical Classification of Diseases and Related Health Problems, 10^th^ Revision). All prescription claims with the H40 code (glaucoma diagnosis) from 2007 to 2020 were extracted. The H40 code contains the following KCD sub-codes: H40.1 (primary open-angle glaucoma), H40.2 (primary angle-closure glaucoma), H40.3 (glaucoma secondary to eye trauma), H40.4 (glaucoma secondary to eye inflammation), H40.5 (glaucoma secondary to other eye disorders), H40.6 (glaucoma secondary to drugs), H40.8 (other glaucoma), H40.9 (unspecified glaucoma), and H42.0 (glaucoma in diseases classified elsewhere).

Prescribed glaucoma eye drops were classified as follows based on their active ingredients: alpha agonist eye drops (A), beta blocker eye drops (B), carbonic anhydrase inhibitor eye drops (C), pilocarpine eye drops (M), prostaglandin analog eye drops (P), alpha agonist/beta blocker fixed-combination eye drops (AB), alpha agonist/carbonic anhydrase inhibitor fixed-combination eye drops (AC), carbonic anhydrase inhibitor/beta blocker fixed-combination eye drops (CB), and prostaglandin analog/beta blocker fixed-combination eye drops (PB).

Theoretically, 511 types of glaucoma eye drop prescription are possible: 9 types for each eye drop alone, 36 types for two eye drop combinations, 84 types for three eye drop combinations, 126 types for four eye drop combinations, 126 types for five eye drop combinations, 84 types for six eye drop combinations, 36 types for seven eye drop combinations, 9 types for eight eye drop combinations, and 1 type for all glaucoma eye drop combinations.

### Patients diagnosed with glaucoma and prescribed glaucoma eye drops

Study participants were grouped by sex and age per year. The prevalence of patients diagnosed with glaucoma and prescribed glaucoma eye drops was calculated based on national population and housing census data from Statistics Korea (https://www.census.go.kr).

Linear regression analysis was used to investigate temporal trends in the number and prevalence of patients diagnosed with glaucoma and prescribed glaucoma eye drops.

### Types of glaucoma eye drop prescription

We investigated the types of glaucoma eye drop prescription for each year. A weight was assigned to each prescription using the reciprocal of the total number of prescriptions received by the individual in that year. The number of patients who received each type of glaucoma eye drop prescription was calculated by summing the weights for each year.

Linear regression analysis was performed to analyze temporal trends in the number of patients who received each type of glaucoma eye drop prescription and their percentage of the total.

### Glaucoma eye drops

We calculated the number of patients prescribed each glaucoma eye drop by summing the weights of the glaucoma eye drop prescriptions that include the respective eye drops.

Linear regression analysis was performed to analyze temporal trends in the number of patients prescribed each glaucoma eye drop and their percentage of the total. In addition, the number of glaucoma eye drops prescribed per patient over 14 years was calculated.

### Active ingredients of glaucoma eye drops

We calculated the number of patients prescribed each active ingredient of glaucoma eye drop by summing the weights of the glaucoma eye drop prescriptions that include the respective active ingredients.

Linear regression analysis was performed to analyze temporal trends in the number of patients prescribed each active ingredient of glaucoma eye drop and their percentage of the total. In addition, the number of active ingredients prescribed per patient over 14 years was calculated.

### Types of glaucoma eye drop prescription according to sex and age

We grouped patients with glaucoma eye drop prescription in 2020 according to sex and age. We analyzed the differences in types of glaucoma eye drop prescriptions based on sex using multivariate logistic regression analysis adjusted for age and examined the changes in prescriptions with increasing age by linear regression analysis.

### Data access and analysis

All data access and statistical analyses were conducted using RStudio version 1.1.463 (RStudio, PBC) through HIRA’s remote access statistical analysis system, *P* < 0.05 was considered statistically significant.

## Results

### Patients diagnosed with glaucoma and prescribed glaucoma eye drops

This study included the data of all patients diagnosed with glaucoma and prescribed glaucoma eye drops in the HIRA database between 2007 and 2020. The number of patients per year from 2007 to 2020 was 211,356; 233,416; 260,746; 291,823; 328,972; 366,743; 406,505; 445,374; 493,051; 536,762; 588,143; 638,471; 701,457; and 729,871; respectively (Table 1 and S1 Fig).

**Table 1:** Number of patients prescribed glaucoma eye drops between 2007 and 2020 in Korea.

Sex	Year																												
Age group	2007		2008		2009		2010		2011		2012		2013		2014		2015		2016		2017		2018		2019		2020		
Men																													
0–9	474	(0.2)	456	(0.2)	469	(0.2)	424	(0.1)	396	(0.1)	430	(0.1)	487	(0.1)	459	(0.1)	528	(0.1)	509	(0.1)	540	(0.1)	585	(0.1)	587	(0.1)	567	(0.1)
10–19	2,053	(1.0)	2,161	(0.9)	2,254	(0.9)	2,472	(0.8)	2,465	(0.7)	2,759	(0.8)	2,752	(0.7)	2,801	(0.6)	2,704	(0.5)	2,532	(0.5)	2,413	(0.4)	2,469	(0.4)	2,314	(0.3)	1,861	(0.3)
20–29	5,148	(2.4)	5,257	(2.3)	5,517	(2.1)	5,843	(2.0)	6,413	(1.9)	7,432	(2.0)	8,107	(2.0)	7,903	(1.8)	7,924	(1.6)	7,403	(1.4)	7,708	(1.3)	7,720	(1.2)	7,958	(1.1)	7,872	(1.1)
30–39	9,768	(4.6)	10,165	(4.4)	10,696	(4.1)	11,337	(3.9)	12,633	(3.8)	13,885	(3.8)	14,686	(3.6)	15,085	(3.4)	15,418	(3.1)	15,449	(2.9)	16,014	(2.7)	16,133	(2.5)	16,595	(2.4)	16,137	(2.2)
40–49	16,749	(7.9)	17,768	(7.6)	19,400	(7.4)	20,984	(7.2)	22,790	(6.9)	24,909	(6.8)	26,860	(6.6)	29,448	(6.6)	32,222	(6.5)	33,996	(6.3)	36,638	(6.2)	38,542	(6.0)	40,058	(5.7)	40,301	(5.5)
50–59	22,020	(10.4)	24,097	(10.3)	26,694	(10.2)	30,241	(10.4)	34,817	(10.6)	39,295	(10.7)	42,920	(10.6)	46,844	(10.5)	51,780	(10.5)	55,732	(10.4)	60,541	(10.3)	65,050	(10.2)	70,746	(10.1)	72,909	(10.0)
60–69	27,650	(13.1)	30,845	(13.2)	34,439	(13.2)	38,619	(13.2)	42,526	(12.9)	45,955	(12.5)	50,065	(12.3)	55,330	(12.4)	62,495	(12.7)	70,124	(13.1)	78,000	(13.3)	85,376	(13.4)	94,885	(13.5)	101,420	(13.9)
70–79	20,130	(9.5)	23,412	(10.0)	27,539	(10.6)	31,750	(10.9)	36,735	(11.2)	41,886	(11.4)	48,405	(11.9)	54,218	(12.2)	60,556	(12.3)	65,613	(12.2)	72,880	(12.4)	81,082	(12.7)	90,309	(12.9)	94,492	(13.0)
80–89	4,807	(2.3)	5,801	(2.5)	7,055	(2.7)	8,419	(2.9)	9,754	(3.0)	11,338	(3.1)	13,349	(3.3)	15,629	(3.5)	18,711	(3.8)	21,784	(4.1)	25,503	(4.3)	29,409	(4.6)	34,387	(4.9)	37,498	(5.1)
90-	232	(0.1)	283	(0.1)	380	(0.1)	479	(0.2)	579	(0.2)	712	(0.2)	890	(0.2)	1,068	(0.2)	1,276	(0.3)	1,412	(0.3)	1,622	(0.3)	1,943	(0.3)	2,259	(0.3)	2,582	(0.4)
Women																												
0–9	346	(0.2)	304	(0.1)	338	(0.1)	327	(0.1)	300	(0.1)	368	(0.1)	420	(0.1)	456	(0.1)	622	(0.1)	562	(0.1)	630	(0.1)	521	(0.1)	548	(0.1)	589	(0.1)
10–19	1,447	(0.7)	1,495	(0.6)	1,464	(0.6)	1,622	(0.6)	1,697	(0.5)	1,879	(0.5)	1,987	(0.5)	2,002	(0.4)	1,917	(0.4)	1,739	(0.3)	1,645	(0.3)	1,489	(0.2)	1,478	(0.2)	1,395	(0.2)
20–29	4,551	(2.2)	4,619	(2.0)	5,100	(2.0)	5,387	(1.8)	5,985	(1.8)	6,744	(1.8)	7,703	(1.9)	7,244	(1.6)	6,893	(1.4)	6,317	(1.2)	6,410	(1.1)	6,314	(1.0)	6,248	(0.9)	6,325	(0.9)
30–39	5,849	(2.8)	6,041	(2.6)	6,379	(2.4)	6,985	(2.4)	7,940	(2.4)	8,963	(2.4)	9,603	(2.4)	9,802	(2.2)	10,032	(2.0)	10,118	(1.9)	10,595	(1.8)	10,666	(1.7)	11,012	(1.6)	10,643	(1.5)
40–49	9,287	(4.4)	10,008	(4.3)	10,623	(4.1)	11,431	(3.9)	12,723	(3.9)	14,160	(3.9)	15,507	(3.8)	17,264	(3.9)	19,137	(3.9)	20,737	(3.9)	22,887	(3.9)	24,620	(3.9)	26,160	(3.7)	26,851	(3.7)
50–59	16,050	(7.6)	17,409	(7.5)	19,005	(7.3)	21,434	(7.3)	24,984	(7.6)	28,087	(7.7)	30,817	(7.6)	33,963	(7.6)	37,495	(7.6)	41,015	(7.6)	44,384	(7.5)	47,963	(7.5)	52,474	(7.5)	54,419	(7.5)
60–69	28,350	(13.4)	30,983	(13.3)	34,267	(13.1)	37,265	(12.8)	40,929	(12.4)	43,685	(11.9)	46,957	(11.6)	50,857	(11.4)	56,819	(11.5)	63,981	(11.9)	69,896	(11.9)	75,639	(11.8)	83,961	(12.0)	88,555	(12.1)
70–79	28,121	(13.3)	32,216	(13.8)	37,014	(14.2)	41,996	(14.4)	47,829	(14.5)	53,929	(14.7)	61,075	(15.0)	67,310	(15.1)	73,981	(15.0)	79,597	(14.8)	86,006	(14.6)	92,902	(14.6)	101,683	(14.5)	103,483	(14.2)
80–89	7,799	(3.7)	9,483	(4.1)	11,370	(4.4)	13,935	(4.8)	16,440	(5.0)	19,075	(5.2)	22,259	(5.5)	25,786	(5.8)	30,310	(6.1)	35,520	(6.6)	40,748	(6.9)	46,267	(7.2)	53,016	(7.6)	56,680	(7.8)
90-	525	(0.2)	613	(0.3)	742	(0.3)	872	(0.3)	1,037	(0.3)	1,251	(0.3)	1,654	(0.4)	1,905	(0.4)	2,231	(0.5)	2,622	(0.5)	3,083	(0.5)	3,781	(0.6)	4,779	(0.7)	5,292	(0.7)
Total	211,356	(100.0)	233,416	(100.0)	260,745	(100.0)	291,822	(100.0)	328,972	(100.0)	366,742	(100.0)	406,503	(100.0)	445,374	(100.0)	493,051	(100.0)	536,762	(100.0)	588,143	(100.0)	638,471	(100.0)	701,457	(100.0)	729,871	(100.0)

The number of patients significantly increased over time for both sexes and across all age groups, except for the 10–19-year age group in both sexes (S1 Table). The total number of patients increased by 41,362 patients/year (*P* < 0.001). The 70–79-year age group showed the highest increase in glaucoma eye drop prescription in both sexes (5,887 patients/year [*P* < 0.001] in men and 6,110 patients/year [*P* < 0.001] in women).

The prevalence of patients, calculated based on the entire Korean population, significantly increased over time, with 0.449%, 0.496%, 0.554%, 0.608%, 0.685%, 0.764%, 0.847%, 0.928%, 0.965%, 1.047%, 1.144%, 1.237%, 1.355%, and 1.408% from 2007 to 2020, respectively (0.075%/year, *P* < 0.001) (S2 Table). Across both sexes, the 80–89-year age group showed the highest increase (0.284%/year [*P* < 0.001] in men and 0.256%/year [*P* < 0.001] in women).

### Types of glaucoma eye drop prescription

We investigated the types of glaucoma eye drop prescription per year, and P was the most frequently prescribed type in all the years (Table 2 and S2 and S3 Figs). In 2007 and 2008, B was the second most frequently prescribed type, whereas CB became the second most frequently prescribed type from 2009 onwards.

**Table 2:** Number of patients who received each type of glaucoma eye drop prescription between 2007 and 2020 in Korea.

	Year																											
Type	2007		2008		2009		2010		2011		2012		2013		2014		2015		2016		2017		2018		2019		2020	
P	57,012	(27.0)	63,231	(27.1)	69,966	(26.8)	79,373	(27.2)	90,053	(27.4)	99,182	(27.0)	109,396	(26.9)	118,420	(26.6)	129,800	(26.3)	141,404	(26.3)	153,674	(26.1)	165,441	(25.9)	181,660	(25.9)	187,601	(25.7)
CB	31,533	(14.9)	34,078	(14.6)	39,784	(15.3)	42,370	(14.5)	62,804	(19.1)	79,650	(21.7)	94,580	(23.3)	106,391	(23.9)	121,043	(24.5)	126,126	(23.5)	137,854	(23.4)	152,380	(23.9)	171,126	(24.4)	182,125	(25.0)
P+CB	12,767	(6.0)	13,569	(5.8)	15,304	(5.9)	16,179	(5.5)	21,499	(6.5)	25,285	(6.9)	29,724	(7.3)	34,040	(7.6)	38,508	(7.8)	41,302	(7.7)	45,115	(7.7)	49,936	(7.8)	55,108	(7.9)	59,231	(8.1)
A	19,273	(9.1)	21,277	(9.1)	23,690	(9.1)	25,688	(8.8)	29,086	(8.8)	32,825	(9.0)	36,398	(9.0)	40,715	(9.1)	45,793	(9.3)	48,719	(9.1)	51,380	(8.7)	52,493	(8.2)	56,199	(8.0)	54,346	(7.4)
AB	1,931	(0.9)	9,268	(4.0)	12,465	(4.8)	15,053	(5.2)	17,495	(5.3)	19,694	(5.4)	21,878	(5.4)	25,579	(5.7)	29,705	(6.0)	36,101	(6.7)	42,663	(7.3)	47,467	(7.4)	51,786	(7.4)	51,674	(7.1)
B	35,791	(16.9)	34,731	(14.9)	34,877	(13.4)	34,477	(11.8)	34,174	(10.4)	32,767	(8.9)	32,372	(8.0)	32,216	(7.2)	33,707	(6.8)	32,829	(6.1)	34,718	(5.9)	36,941	(5.8)	39,482	(5.6)	38,655	(5.3)
P+CB+A	7,170	(3.4)	8,253	(3.5)	9,446	(3.6)	10,320	(3.5)	13,149	(4.0)	15,272	(4.2)	17,903	(4.4)	20,657	(4.6)	23,371	(4.7)	25,376	(4.7)	28,029	(4.8)	31,085	(4.9)	33,691	(4.8)	36,231	(5.0)
CB+A	9,142	(4.3)	10,218	(4.4)	11,677	(4.5)	12,290	(4.2)	15,184	(4.6)	17,190	(4.7)	19,429	(4.8)	22,020	(4.9)	24,104	(4.9)	25,459	(4.7)	27,681	(4.7)	29,750	(4.7)	32,205	(4.6)	33,746	(4.6)
PB	6,078	(2.9)	6,240	(2.7)	8,913	(3.4)	9,504	(3.3)	9,161	(2.8)	8,241	(2.2)	7,737	(1.9)	7,332	(1.6)	6,990	(1.4)	7,928	(1.5)	11,747	(2.0)	14,951	(2.3)	18,300	(2.6)	21,554	(3.0)
P+AB	409	(0.2)	2,224	(1.0)	3,073	(1.2)	3,906	(1.3)	4,849	(1.5)	5,547	(1.5)	5,934	(1.5)	6,598	(1.5)	7,502	(1.5)	8,697	(1.6)	9,745	(1.7)	10,781	(1.7)	11,769	(1.7)	12,036	(1.6)
P+A	5,018	(2.4)	5,209	(2.2)	5,317	(2.0)	5,495	(1.9)	6,536	(2.0)	7,426	(2.0)	8,165	(2.0)	8,823	(2.0)	9,327	(1.9)	9,320	(1.7)	9,644	(1.6)	9,826	(1.5)	10,207	(1.5)	10,326	(1.4)
C	3,433	(1.6)	3,154	(1.4)	3,409	(1.3)	11,276	(3.9)	3,008	(0.9)	3,002	(0.8)	3,322	(0.8)	3,601	(0.8)	3,869	(0.8)	4,315	(0.8)	4,859	(0.8)	5,354	(0.8)	6,058	(0.9)	6,115	(0.8)
AC																	17	(0.0)	7,594	(1.4)	6,727	(1.1)	5,427	(0.8)	4,811	(0.7)	4,502	(0.6)
P+B	6,439	(3.0)	6,320	(2.7)	6,330	(2.4)	6,149	(2.1)	5,608	(1.7)	5,076	(1.4)	4,810	(1.2)	4,612	(1.0)	4,591	(0.9)	4,461	(0.8)	4,410	(0.7)	4,518	(0.7)	4,543	(0.6)	4,473	(0.6)
PB+AC																			575	(0.1)	1,590	(0.3)	2,430	(0.4)	3,210	(0.5)	4,404	(0.6)
M	2,639	(1.2)	2,616	(1.1)	2,449	(0.9)	2,324	(0.8)	2,245	(0.7)	2,200	(0.6)	2,066	(0.5)	1,886	(0.4)	1,858	(0.4)	1,870	(0.3)	2,002	(0.3)	2,125	(0.3)	2,284	(0.3)	2,483	(0.3)
P+C	1,303	(0.6)	1,350	(0.6)	1,418	(0.5)	3,145	(1.1)	1,613	(0.5)	1,538	(0.4)	1,588	(0.4)	1,709	(0.4)	1,783	(0.4)	1,819	(0.3)	1,965	(0.3)	2,074	(0.3)	2,253	(0.3)	2,475	(0.3)
A+PB	953	(0.5)	959	(0.4)	1,253	(0.5)	1,332	(0.5)	1,323	(0.4)	1,244	(0.3)	1,158	(0.3)	1,059	(0.2)	1,063	(0.2)	1,055	(0.2)	1,271	(0.2)	1,489	(0.2)	1,785	(0.3)	2,048	(0.3)
P+AC																	2	(0.0)	1,453	(0.3)	1,865	(0.3)	1,989	(0.3)	1,961	(0.3)	1,988	(0.3)
P+AB+C	40	(0.0)	338	(0.1)	613	(0.2)	822	(0.3)	896	(0.3)	933	(0.3)	944	(0.2)	1,022	(0.2)	1,076	(0.2)	1,152	(0.2)	1,232	(0.2)	1,336	(0.2)	1,408	(0.2)	1,455	(0.2)
C+AB	42	(0.0)	471	(0.2)	662	(0.3)	884	(0.3)	824	(0.3)	762	(0.2)	797	(0.2)	855	(0.2)	925	(0.2)	943	(0.2)	1,093	(0.2)	1,237	(0.2)	1,276	(0.2)	1,349	(0.2)
CB+AB	18	(0.0)	110	(0.0)	190	(0.1)	216	(0.1)	337	(0.1)	432	(0.1)	560	(0.1)	541	(0.1)	627	(0.1)	750	(0.1)	757	(0.1)	869	(0.1)	1,017	(0.1)	992	(0.1)
CB+PB	237	(0.1)	222	(0.1)	346	(0.1)	370	(0.1)	511	(0.2)	493	(0.1)	453	(0.1)	407	(0.1)	402	(0.1)	399	(0.1)	497	(0.1)	591	(0.1)	760	(0.1)	945	(0.1)
P+CB+AB	8	(0.0)	62	(0.0)	82	(0.0)	111	(0.0)	186	(0.1)	242	(0.1)	313	(0.1)	365	(0.1)	453	(0.1)	510	(0.1)	582	(0.1)	653	(0.1)	766	(0.1)	848	(0.1)
PB+C	407	(0.2)	405	(0.2)	491	(0.2)	668	(0.2)	553	(0.2)	465	(0.1)	437	(0.1)	412	(0.1)	369	(0.1)	360	(0.1)	415	(0.1)	502	(0.1)	639	(0.1)	809	(0.1)
Others	9,712	(4.6)	9,113	(3.9)	8,990	(3.4)	9,870	(3.4)	7,879	(2.4)	7,277	(2.0)	6,543	(1.6)	6,113	(1.4)	6,166	(1.3)	6,243	(1.2)	6,627	(1.1)	6,829	(1.1)	7,152	(1.0)	7,459	(1.0)
Total	211,356	(100.0)	233,416	(100.0)	260,746	(100.0)	291,823	(100.0)	328,972	(100.0)	366,743	(100.0)	406,505	(100.0)	445,374	(100.0)	493,051	(100.0)	536,762	(100.0)	588,143	(100.0)	638,471	(100.0)	701,457	(100.0)	729,871	(100.0)

P = prostaglandin analog eye drops, CB = carbonic anhydrase inhibitor/beta blocker fixed-combination eye drops, A = alpha agonist eye drops, AB = alpha agonist/beta blocker fixed-combination eye drops, B = beta blocker eye drops, PB = prostaglandin analog/beta blocker fixed-combination eye drops, C = carbonic anhydrase inhibitor eye drops, AC = alpha agonist/carbonic anhydrase inhibitor fixed-combination eye drops, M = pilocarpine eye drops

In 2020, the 10 most frequently received types of glaucoma eye drop prescription were P (187,601 patients, 25.7%), CB (182,125 patients, 25.0%), P+CB (59,231 patients, 8.1%), A (54,346 patients, 7.4%), AB (51,674 patients, 7.1%), B (38,655 patients, 5.3%), P+CB+A (36,231 patients, 5.0%), CB+A (33,746 patients, 4.6%), PB (21,554 patients, 3.0%), and P+AB (12,036 patients, 1.6%).

From 2007 to 2020, the number of patients who received all 10 most frequent glaucoma eye drop prescription types, except B (*P* = 0.120), significantly increased (S3 Table). The percentage of patients increased significantly in the CB (0.900%/year, *P* < 0.001), P+CB (0.200%/year, *P* < 0.001), AB (0.364%/year, *P* < 0.001), P+CB+A (0.131%/year, *P* < 0.001), CB+A (0.028%/year, *P* = 0.037), and P+AB (0.076%/year, *P* < 0.001) groups but decreased significantly in the P (-0.117%/year, *P* < 0.001), A (-0.087%/year, *P* = 0.005), and B (-0.860%/year, *P* < 0.001) groups.

### Glaucoma eye drops

Among the nine glaucoma eye drop types, P was the most frequently prescribed in all the years, followed by CB (Table 3 and S4 and S5 Figs). In 2007, B was the third most frequently prescribed, whereas A became the third most frequently prescribed glaucoma eye drop from 2008 onwards.

**Table 3:** Number of patients prescribed each glaucoma eye drop between 2007 and 2020 in Korea.

	Year																											
Eye drops	2007		2008		2009		2010		2011		2012		2013		2014		2015		2016		2017		2018		2019		2020	
P	93068	(44.0)	103425	(44.3)	114417	(43.9)	128778	(44.1)	147064	(44.7)	163036	(44.5)	181099	(44.6)	198499	(44.6)	218760	(44.4)	237877	(44.3)	258769	(44.0)	280255	(43.9)	306095	(43.6)	319418	(43.8)
CB	63370	(30.0)	69024	(29.6)	79512	(30.5)	84347	(28.9)	116449	(35.4)	141221	(38.5)	165382	(40.7)	186535	(41.9)	210663	(42.7)	222212	(41.4)	243083	(41.3)	267956	(42.0)	297428	(42.4)	317126	(43.4)
A	46648	(22.1)	50714	(21.7)	56235	(21.6)	61078	(20.9)	69576	(21.1)	78014	(21.3)	86810	(21.4)	97034	(21.8)	107528	(21.8)	113666	(21.2)	121897	(20.7)	128541	(20.1)	138066	(19.7)	140702	(19.3)
AB	2557	(1.2)	12973	(5.6)	17795	(6.8)	21679	(7.4)	25269	(7.7)	28238	(7.7)	31027	(7.6)	35564	(8.0)	40956	(8.3)	48928	(9.1)	56963	(9.7)	63254	(9.9)	69041	(9.8)	69424	(9.5)
B	48171	(22.8)	46162	(19.8)	45827	(17.6)	44732	(15.3)	43343	(13.2)	41020	(11.2)	39928	(9.8)	39397	(8.8)	40776	(8.3)	39671	(7.4)	41456	(7.0)	43915	(6.9)	46649	(6.7)	45777	(6.3)
PB	8288	(3.9)	8428	(3.6)	11852	(4.5)	12852	(4.4)	12484	(3.8)	11217	(3.1)	10482	(2.6)	9841	(2.2)	9482	(1.9)	11003	(2.0)	16369	(2.8)	20903	(3.3)	25866	(3.7)	31298	(4.3)
C	8070	(3.8)	8262	(3.5)	9114	(3.5)	20925	(7.2)	9075	(2.8)	8683	(2.4)	8986	(2.2)	9528	(2.1)	10024	(2.0)	10340	(1.9)	11241	(1.9)	12202	(1.9)	13375	(1.9)	13947	(1.9)
AC																	21	(0.0)	10041	(1.9)	10812	(1.8)	10603	(1.7)	10821	(1.5)	11874	(1.6)
M	6247	(3.0)	6068	(2.6)	5744	(2.2)	5308	(1.8)	5148	(1.6)	4926	(1.3)	4352	(1.1)	3777	(0.8)	3712	(0.8)	3677	(0.7)	3935	(0.7)	4028	(0.6)	4087	(0.6)	4289	(0.6)
Total	211356	(130.8)	233416	(130.7)	260746	(130.6)	291823	(130.1)	328972	(130.2)	366743	(129.9)	406505	(129.9)	445374	(130.3)	493051	(130.2)	536762	(129.9)	588143	(130.0)	638471	(130.3)	701457	(129.9)	729871	(130.7)

P = prostaglandin analog eye drops, CB = carbonic anhydrase inhibitor/beta blocker fixed-combination eye drops, A = alpha agonist eye drops, AB = alpha agonist/beta blocker fixed-combination eye drops, B = beta blocker eye drops, PB = prostaglandin analog/beta blocker fixed-combination eye drops, C = carbonic anhydrase inhibitor eye drops, AC = alpha agonist/carbonic anhydrase inhibitor fixed-combination eye drops, M = pilocarpine eye drops

From 2007 to 2020, a significant increase was observed in the number of patients prescribed P (18050.0 patients/year, *P* < 0.001), CB (20610.0 patients/year, *P* < 0.001), A (7866.0 patients/year, *P* < 0.001), AB (5062.0 patients/year, *P* < 0.001), and PB (1283.0 patients/year, *P* = 0.001) (S4 Table). However, no significant changes were observed in the number of patients prescribed B (-188.0 patients/year, *P* = 0.356), C (227.6 patients/year, *P* = 0.333), and AC (1754.2 patients/year, *P* = 0.095), but a significant decrease was observed in the number of patients prescribed M (-184.3 patients/year, *P* < 0.001).

The percentage of patients increased significantly in the CB (1.210%/year, *P* < 0.001) and AB (0.457%/year, *P* < 0.001) groups. However, no significant changes were observed in the P (-0.030%/year, *P* = 0.171), PB (-0.065%/year, *P* = 0.282), and AC (0.199%/year, *P* = 0.284) groups, but significant decreases were observed in the A (-0.156%/year, *P* < 0.001), B (-1.213%/year, *P* < 0.001), C (-0.218%/year, *P* = 0.014), and M (-0.179%/year, *P* < 0.001) groups.

The proportion of single-ingredient glaucoma eye drops from 2007 to 2020 decreased from 73.2% to 70.4%, 67.9%, 68.7%, 64.0%, 62.1%, 60.8%, 60.0%, 59.3%, 59.0%, 58.0%, 57.1%, 56.4%, and finally to 55.6% (-1.290%/year, *P* < 0.001) per year, respectively. In contrast, the proportion of fixed-combination glaucoma eye drops from 2007 to 2020 increased from 26.8% to 29.6%, 32.1%, 31.3%, 36.0%, 37.9%, 39.2%, 40.0%, 40.7%, 41.0%, 42.0%, 42.9%, 43.6%, and finally to 44.4% (1.291%/year, *P* < 0.001) per year, respectively.

No significant difference was observed in the number of glaucoma eye drops prescribed per patient over the 14-year period (-0.00030/year, *P* = 0.167), with an average of 1.302.

### Active ingredients of glaucoma eye drops

Among the five types of active ingredients for glaucoma eye drops, beta blockers were the most frequently prescribed in all the years, followed by prostaglandin analogs, carbonic anhydrase inhibitors, alpha agonists, and pilocarpines (Table 4 and S6 and S7 Figs).

**Table 4:** Number of patients prescribed each active ingredient of glaucoma eye drop between 2007 and 2020 in Korea.

	Year																																								
Active ingredients	2007		2008			2009			2010			2011			2012			2013			2014			2015			2016			2017			2018			2019			2020		
Beta blocker	122386	(57.9)		136587	(58.5)		154986	(59.4)		163611	(56.1)		197545	(60.0)		221696	(60.4)		246820	(60.7)		271337	(60.9)		301877	(61.2)		321814	(60.0)		357870	(60.8)		396027	(62.0)		438984	(62.6)		463625	(63.5)	
Prostaglandin analog	101357	(48.0)		111853	(47.9)		126269	(48.4)		141631	(48.5)		159548	(48.5)		174252	(47.5)		191581	(47.1)		208340	(46.8)		228241	(46.3)		248880	(46.4)		275137	(46.8)		301157	(47.2)		331961	(47.3)		350716	(48.1)	
Carbonic anhydrase inhibitor	71439	(33.8)		77287	(33.1)		88626	(34.0)		105272	(36.1)		125524	(38.2)		149904	(40.9)		174369	(42.9)		196064	(44.0)		220708	(44.8)		242593	(45.2)		265135	(45.1)		290761	(45.5)		321624	(45.9)		342947	(47.0)	
Alpha agonist	49206	(23.3)		63687	(27.3)		74029	(28.4)		82757	(28.4)		94845	(28.8)		106252	(29.0)		117838	(29.0)		132598	(29.8)		148505	(30.1)		172634	(32.2)		189672	(32.2)		202398	(31.7)		217928	(31.1)		222000	(30.4)	
Pilocarpine	6247	(3.0)		6068	(2.6)		5744	(2.2)		5308	(1.8)		5148	(1.6)		4926	(1.3)		4352	(1.1)		3777	(0.8)		3712	(0.8)		3677	(0.7)		3935	(0.7)		4028	(0.6)		4087	(0.6)		4289	(0.6)	
Total	211356	(165.9)		233416	(169.4)		260746	(172.4)		291823	(170.8)		328972	(177.1)		366743	(179.2)		406505	(180.8)		445374	(182.3)		493051	(183.2)		536762	(184.4)		588143	(185.6)		638471	(187.1)		701457	(187.4)		729871	(189.6)	

From 2007 to 2020, the number of patients prescribed all active ingredients, except pilocarpine (-184.3 patients/year, *P* < 0.001), significantly increased (S5 Table).

The percentage of patients prescribed beta blockers (0.388%/year, *P* < 0.001), carbonic anhydrase inhibitors (1.157%/year, *P* < 0.001), and alpha agonists (0.467%/year, *P* < 0.001) increased significantly. However, no significant change was observed in the percentage of patients prescribed prostaglandin analogs (-0.096%/year, *P* = 0.056), but a significant decrease was observed in the percentage of patients prescribed pilocarpine (-0.156%/year, *P* < 0.001).

There was a significant increase in the number of active ingredients prescribed per patient over the 14-year period (0.01737/year, *P* < 0.001), from 1.659 in 2007 to 1.896 in 2020.

### Types of glaucoma eye drop prescription according to sex and age in 2020

In 2020, the most frequently given types of glaucoma eye drop prescription for men were CB (95,143 patients, 25.3%), P (89.812 patients, 23.9%), and P+CB (33,044 patients, 8.8%), whereas those for women were P (97,689 patients, 27.6%), CB (86,982 patients, 24.6%), and P+CB (26,187 patients, 7.4%) (Table 5 and S6 and S7 Tables). Among the 10 most frequently given types of glaucoma eye drop prescription in 2020, P+CB, P+CB+A, CB+A, PB, and P+AB were significantly more for men than for women, whereas P, A, AB, and B were significantly more for women than for men (all *P* < 0.001).

**Table 5:** Number of patients who received each type of glaucoma eye drop prescription according to sex in 2020.

Type	Men		Women		*P* value*	*P* value†
P	89,912	(23.9)	97,689	(27.6)	<0.001	<0.001
CB	95,143	(25.3)	86,982	(24.6)	<0.001	0.061
P+CB	33,044	(8.8)	26,187	(7.4)	<0.001	<0.001
A	26,085	(6.9)	28,260	(8.0)	<0.001	<0.001
AB	25,425	(6.8)	26,249	(7.4)	<0.001	<0.001
B	16,036	(4.3)	22,619	(6.4)	<0.001	<0.001
P+CB+A	23,158	(6.2)	13,073	(3.7)	<0.001	<0.001
CB+A	20,380	(5.4)	13,367	(3.8)	<0.001	<0.001
PB	11,372	(3.0)	10,182	(2.9)	<0.001	<0.001
P+AB	6,794	(1.8)	5,242	(1.5)	<0.001	<0.001
P+A	5,673	(1.5)	4,653	(1.3)	<0.001	<0.001
C	2,897	(0.8)	3,218	(0.9)	<0.001	<0.001
AC	2,318	(0.6)	2,184	(0.6)	0.979	0.467
P+B	2,154	(0.6)	2,319	(0.7)	<0.001	0.021
PB+AC	2,835	(0.8)	1,569	(0.4)	<0.001	<0.001
M	1,150	(0.3)	1,333	(0.4)	<0.001	<0.001
P+C	1,186	(0.3)	1,289	(0.4)	<0.001	0.026
PB+A	1,228	(0.3)	820	(0.2)	<0.001	<0.001
P+AC	1,164	(0.3)	825	(0.2)	<0.001	<0.001
P+C+AB	897	(0.2)	558	(0.2)	<0.001	<0.001
C+AB	728	(0.2)	621	(0.2)	0.067	0.229
CB+AB	567	(0.2)	425	(0.1)	<0.001	0.001
PB+CB	547	(0.1)	398	(0.1)	<0.001	<0.001
P+CB+AB	530	(0.1)	318	(0.1)	<0.001	<0.001
PB+C	419	(0.1)	390	(0.1)	0.788	0.712
Others	3,998	(1.1)	3,461	(1.0)	<0.001	<0.001
Total	375,639	(100.0)	354,232	(100.0)		

P = prostaglandin analog eye drops, CB = carbonic anhydrase inhibitor/beta blocker fixed-combination eye drops, A = alpha agonist eye drops, AB = alpha agonist/beta blocker fixed-combination eye drops, B = beta blocker eye drops, PB = prostaglandin analog/beta blocker fixed-combination eye drops, C = carbonic anhydrase inhibitor eye drops, AC = alpha agonist/carbonic anhydrase inhibitor fixed-combination eye drops, M = pilocarpine eye drops

*Univariate logistic regression analysis.

†Multivariate logistic regression analysis adjusting for age.

In 2020, the most frequently given types of glaucoma eye drop prescription for the 0–9-year age group were CB (303 patients, 26.3%), A (289 patients, 25%), and B (229 patients, 19.8%), whereas for the 90-year age group, the order was P (2,400 patients, 30.5%), BC (1,355 patients, 17.2%), and P+BC (678 patients, 8.6%) (Table 6 and S8 Fig). The percentage of patients increased significantly with age in the P (2.834%/10 years, *P* < 0.001), PB (0.284%/10 years, *P* < 0.001), and P+AB (0.161%/10 years, *P* < 0.001) groups and decreased significantly in the CB (-2.067%/10 years, *P* = 0.006) and A (-1.267%/10 years, *P* = 0.025) groups (S8 Table).

**Table 6:** Number of patients who received each type of glaucoma eye drop prescription according to age group in 2020.

	Age groups																			
Type	0–9 years		10–19 years		20–29 years		30–39 years		40–49 years		50–59 years		60–69 years		70–79 years		80–89 years		90- years	
P	66	(5.7)	362	(11.1)	1,693	(11.9)	4,473	(16.7)	14,667	(21.8)	30,081	(23.6)	49,488	(26.0)	55,993	(28.3)	28,378	(30.1)	2,400	(30.5)
CB	303	(26.3)	1,286	(39.5)	5,643	(39.7)	9,299	(34.7)	19,708	(29.3)	35,020	(27.5)	47,275	(24.9)	44,016	(22.2)	18,220	(19.3)	1,355	(17.2)
P+CB	121	(10.5)	172	(5.3)	699	(4.9)	1,703	(6.4)	5,247	(7.8)	9,975	(7.8)	15,432	(8.1)	16,715	(8.4)	8,489	(9.0)	678	(8.6)
A	289	(25.0)	369	(11.3)	1,542	(10.9)	2,370	(8.8)	4,690	(7.0)	9,746	(7.7)	13,785	(7.3)	14,295	(7.2)	6,666	(7.1)	595	(7.6)
AB	29	(2.5)	222	(6.8)	1,126	(7.9)	2,024	(7.6)	4,979	(7.4)	9,100	(7.1)	13,879	(7.3)	13,823	(7.0)	6,063	(6.4)	430	(5.5)
B	229	(19.8)	179	(5.5)	739	(5.2)	1,165	(4.3)	3,131	(4.7)	6,080	(4.8)	10,367	(5.5)	11,053	(5.6)	5,206	(5.5)	508	(6.4)
P+CB+A	43	(3.7)	185	(5.7)	671	(4.7)	1,291	(4.8)	3,366	(5.0)	6,480	(5.1)	9,028	(4.8)	9,818	(5.0)	4,899	(5.2)	451	(5.7)
CB+A	19	(1.6)	238	(7.3)	1,036	(7.3)	1,945	(7.3)	3,827	(5.7)	6,526	(5.1)	8,451	(4.4)	7,781	(3.9)	3,607	(3.8)	317	(4.0)
PB	12	(1.0)	24	(0.7)	198	(1.4)	605	(2.3)	1,949	(2.9)	3,625	(2.8)	5,811	(3.1)	6,023	(3.0)	3,063	(3.3)	245	(3.1)
P+AB	1	(0.1)	35	(1.1)	134	(0.9)	345	(1.3)	1,056	(1.6)	1,971	(1.5)	3,075	(1.6)	3,527	(1.8)	1,745	(1.9)	147	(1.9)
P+A	2	(0.2)	36	(1.1)	104	(0.7)	263	(1.0)	798	(1.2)	1,512	(1.2)	2,469	(1.3)	3,156	(1.6)	1,818	(1.9)	167	(2.1)
C	25	(2.2)	51	(1.6)	134	(0.9)	233	(0.9)	694	(1.0)	1,054	(0.8)	1,515	(0.8)	1,625	(0.8)	715	(0.8)	67	(0.8)
AC	0	(0.0)	9	(0.3)	61	(0.4)	163	(0.6)	364	(0.5)	702	(0.6)	1,159	(0.6)	1,353	(0.7)	638	(0.7)	53	(0.7)
P+B	1	(0.1)	6	(0.2)	30	(0.2)	66	(0.2)	276	(0.4)	643	(0.5)	1,126	(0.6)	1,336	(0.7)	898	(1.0)	92	(1.2)
PB+AC	0	(0.0)	15	(0.4)	77	(0.5)	163	(0.6)	439	(0.7)	798	(0.6)	1,142	(0.6)	1,137	(0.6)	565	(0.6)	68	(0.9)
M	2	(0.2)	18	(0.6)	74	(0.5)	104	(0.4)	288	(0.4)	631	(0.5)	722	(0.4)	473	(0.2)	156	(0.2)	14	(0.2)
P+C	7	(0.6)	3	(0.1)	14	(0.1)	48	(0.2)	173	(0.3)	364	(0.3)	620	(0.3)	761	(0.4)	434	(0.5)	53	(0.7)
PB+A	0	(0.0)	0	(0.0)	18	(0.1)	55	(0.2)	184	(0.3)	358	(0.3)	522	(0.3)	568	(0.3)	320	(0.3)	24	(0.3)
P+AC	0	(0.0)	4	(0.1)	19	(0.1)	44	(0.2)	152	(0.2)	282	(0.2)	482	(0.3)	651	(0.3)	320	(0.3)	34	(0.4)
P+C+AB	0	(0.0)	1	(0.0)	14	(0.1)	43	(0.2)	131	(0.2)	265	(0.2)	367	(0.2)	417	(0.2)	204	(0.2)	13	(0.2)
C+AB	0	(0.0)	9	(0.3)	26	(0.2)	68	(0.3)	146	(0.2)	262	(0.2)	365	(0.2)	327	(0.2)	143	(0.2)	4	(0.0)
CB+AB	1	(0.1)	8	(0.2)	28	(0.2)	36	(0.1)	94	(0.1)	188	(0.1)	265	(0.1)	257	(0.1)	105	(0.1)	11	(0.1)
PB+CB	1	(0.1)	0	(0.0)	10	(0.1)	19	(0.1)	85	(0.1)	170	(0.1)	239	(0.1)	263	(0.1)	149	(0.2)	9	(0.1)
P+CB+AB	0	(0.0)	1	(0.0)	13	(0.1)	30	(0.1)	83	(0.1)	146	(0.1)	218	(0.1)	230	(0.1)	115	(0.1)	11	(0.1)
PB+C	0	(0.0)	0	(0.0)	6	(0.0)	29	(0.1)	68	(0.1)	140	(0.1)	213	(0.1)	224	(0.1)	125	(0.1)	5	(0.1)
Others	5	(0.4)	23	(0.7)	88	(0.6)	196	(0.7)	560	(0.8)	1,209	(0.9)	1,958	(1.0)	2,157	(1.1)	1,137	(1.2)	124	(1.6)
Total	1,156	(100.0)	3,256	(100.0)	14,197	(100.0)	26,780	(100.0)	67,152	(100.0)	127,328	(100.0)	189,975	(100.0)	197,975	(100.0)	94,178	(100.0)	7,874	(100.0)

P = prostaglandin analog eye drops, CB = carbonic anhydrase inhibitor/beta blocker fixed-combination eye drops, A = alpha agonist eye drops, AB = alpha agonist/beta blocker fixed-combination eye drops, B = beta blocker eye drops, PB = prostaglandin analog/beta blocker fixed-combination eye drops, C = carbonic anhydrase inhibitor eye drops, AC = alpha agonist/carbonic anhydrase inhibitor fixed-combination eye drops, M = pilocarpine eye drops

## Discussion

This study analyzed the glaucoma eye drops prescribed for glaucoma patients in the entire Korean population from 2007 to 2020 using the HIRA database. We identified the glaucoma eye drops and combinations prescribed to each individual and investigated their long-term trends. Additionally, we analyzed the differences in glaucoma eye drop prescription according to sex and age.

### Participants

We found that the prevalence of patients diagnosed with glaucoma and prescribed glaucoma eye drops increased from 0.449% in 2007 to 1.408% in 2020. The prevalence in the population aged ≥40 years was 2.361% in 2020. The Namil study, an epidemiological study, conducted in Koreas using a defined population reported a 3.5% prevalence of primary open-angle glaucoma in residents aged ≥40 years [11]. Another study using the Korea National Health and Nutrition Examination Survey data from 2008 to 2011 reported a 4.7% prevalence of primary open-angle glaucoma in participants aged ≥40 years and found that only 8% were aware of their disease [12]. This study analyzed the overall diagnosis of glaucoma, not just primary open-angle glaucoma, and there may be glaucoma patients without prescriptions for glaucoma medication. Therefore, it is difficult to directly compare the prevalence with that reported in previous studies. The observed increase in patients across all ages in this study is presumed to be due to the widespread implementation of health checkups and improved awareness of glaucoma due to disease education.

### Types of glaucoma eye drop prescription

The most frequently given type of glaucoma eye drop prescription between 2007 and 2020 was P. However, the proportion of P, A, and B decreased significantly during the study period. Conversely, the proportions of CB, P+CB, AB, P+CB+A, CB+A, and P+AB increased significantly. In summary, the proportion of single-ingredient glaucoma eye drop monotherapy prescriptions is decreasing, whereas that of fixed-combination glaucoma eye drop prescriptions, alone or in combination with other glaucoma eye drops, is increasing. A previous study analyzing the prescription trends of topical glaucoma medications in Australia from 2001 to 2017 reported a decrease in prescribing P and B, which were the most commonly prescribed medications in 2007, particularly after the advent of fixed-combination glaucoma eye drops, especially PB [7].

The advantages of fixed-combination therapy over unfixed therapy are as follows: First, they improve patient adherence. Several studies have reported a decrease in adherence as the number of glaucoma eye drop instillations increase [13–15], and other studies have demonstrated that fixed-combination therapy results in significantly higher adherence than unfixed therapy [16–19]. Second, using a fixed-combination glaucoma eye drop can minimize ocular surface damage by reducing exposure to preservatives [19]. Third, a fixed-combination glaucoma eye drop eliminates the washout effect. Previous studies have reported that fixed-combination glaucoma eye drops with dorzolamide and timolol significantly lower intraocular pressure than concomitant administration [20].

### Glaucoma eye drops

Among the nine types of glaucoma eye drops, P was the most frequently prescribed in all the years, and its proportion did not change significantly. The proportions of other single-ingredient glaucoma eye drops, such as A, B, C, and M, decreased significantly. In contrast, the proportions of fixed-combination glaucoma eye drops, such as CB and AB, increased significantly. This difference reflects an increased preference for fixed-combination glaucoma eye drops.

### Active ingredients of glaucoma eye drops

A, B, and C as single-ingredient glaucoma eye drops showed a significant decrease in proportion, whereas alpha agonists, beta blockers, and carbonic anhydrase inhibitors as active ingredients showed a significant increase in proportion. This increase may be due to the increased use of fixed-combination glaucoma eye drops. Carbonic anhydrase inhibitors showed the fastest increase in proportion, which was considered to be due to the increased prescription of CB.

Over the 14-year period, the number of glaucoma eye drops prescribed per patient did not change, with an average of 1.302; however, the number of active ingredients prescribed per patient increased significantly from 1.659 in 2007 to 1.896 in 2020. It is speculated that the increase in the number of active ingredients prescribed per patient is related to a rise in the severity of glaucoma owing to a growing older adult population. This can be seen from the highest increase in the number of patients in the 70–79-year age group and the highest increase in the prevalence of patients in the 80–89-year age group during the study period. In contrast, the lack of a significant change in the number of glaucoma eye drops prescribed per patient was considered to be due to the increasing prescription of fixed-combination eye drops.

### Types of glaucoma eye drop prescription according to sex and age in 2020

In the multivariate logistic regression analysis with age correction, most types of glaucoma eye drop prescription showed significant differences between men and women. In men, a combination of several glaucoma eye drops, including fixed-combination glaucoma eye drops such as P+BC, P+BC+A, BC+A, and P+AB, was relatively more commonly prescribed. Single glaucoma eye drops, such as P, A, AB, and B, were relatively more commonly prescribed to women. It is speculated that men have more severe glaucoma than women. However, it cannot be definitively concluded because in cases where surgical treatment is received, the use of eye drops may be reduced. Therefore, Additional research is required to draw definitive conclusions.

In the linear regression analysis of the types of glaucoma eye drop prescription in 2020, the proportion of P increased with age, whereas that of CB decreased. Therefore, CB was the most prescribed glaucoma medication for patients below 60 years of age, and P became the most prescribed glaucoma medication for patients aged 60 years and above. CB had a prescription rate of nearly 40%, particularly in the 10–19 and 20–29-year age groups. This is speculated to be due to the relatively lower incidence of eye and eyelid side effects, such as eyelid changes [21–23], eyelid pigmentation [24], and deepening of the eyelid sulcus [25, 26], compared to that of P, making CB prescription preferable in younger age groups.

Most age groups exhibited a similar distribution of given type of glaucoma eye drop prescription to that of adjacent age groups; however, the 0–9-year age group exhibited a distinct difference from the 10–19-year age group. In particular, the proportions of A and B were high at 25.0% and 19.8%, respectively. Beta blockers should be used cautiously owing to the risk of bronchospasms and bradycardia; however, they are generally considered safe for use in pediatric patients with glaucoma [27, 28]. Apraclonidine, an alpha agonist, is known to be relatively safe for use in pediatric patients with glaucoma, compared to brimonidine [29, 30]. Brimonidine is recommended for only patients weighing over 20 kg or > 6 years of age because of its potential to cause severe systemic side effects such as apnea and hypotension [31–33]. In this study, age was not further stratified yearly, and glaucoma eye drops were analyzed by grouping them into categories based on their active ingredients. Therefore, obtaining detailed information on pediatric patients with glaucoma was difficult. Further focused research incorporating clinical data, such as age, weight, and systemic conditions, is required to address this issue.

### Limitations

This study has several limitations. First, there may be discrepancies between the prescribed glaucoma eye drops and actual eye drops used by patients. Various factors, including patient adherence, can influence these discrepancies. However, analyzing prescriptions remains important, as prescriptions reflect the physician’s intended treatment approach based on direct interaction with the patient. Second, if a patient had been prescribed two types of glaucoma eye drops in the previous visit, and one of the eye drops remained unused and was not prescribed in the current visit, this would have been recorded as a change in prescription in this study, although there was no actual change in the intended treatment by the physician.

## Conclusion

We analyzed all prescription data of glaucoma eye drops for patients diagnosed with glaucoma in Korea between 2007 and 2020, and confirmed that P monotherapy was the most prescribed medication during the study period. From 2009, the next most prescribed medication was CB monotherapy, which showed the fastest increase in prescription rate during the study period.

During the study period, the number of glaucoma eye drops prescribed per patient remained constant, whereas the number of active ingredients prescribed per patient increased. This can be seen in the same context as the decrease in the prescription of single-ingredient glaucoma eye drops and increase in the prescription of fixed-combination eye drops.

In addition, we revealed that there are differences in glaucoma eye drop prescription across age groups and based on sex.

Our findings, which demonstrate the long-term prescription trends and current prescription status of glaucoma eye drops, are expected to contribute to the future development of glaucoma medications and establishment of treatment strategies.

## Supporting information

S1 Table Simple linear regression for analyzing the number of patients prescribed glaucoma eye drops according to age and sex. (DOCX)

S2 Table Simple linear regression for analyzing the prevalence of patients (stratified according to age and sex) prescribed glaucoma eye drops per year. (DOCX)

S3 Table Simple linear regression for analyzing the number and percentage of patients who received each type of glaucoma eye drop prescription per year. (DOCX)

S4 Table Simple linear regression for analyzing the number and percentage of patients prescribed each glaucoma eye drop per year. (DOCX)

S5 Table Simple linear regression for analyzing the number and percentage of patients prescribed each active ingredient of glaucoma eye drop per year. (DOCX)

S6 Table Number of patients (men) who received each type of glaucoma eye drop prescription according to age group in 2020. (DOCX)

S7 Table Number of patients (women) who received each type of glaucoma eye drop prescription according to age group in 2020. (DOCX)

S8 Table Simple linear regression for analyzing the percentage of patients who received each type of glaucoma eye drop prescription according to age group in 2020. (DOCX)

S1 Fig Number of patients prescribed glaucoma eye drops in Korea. (TIFF)

S2 Fig Number of patients who received each type of glaucoma eye drop prescription. P, prostaglandin analog eye drops; CB, carbonic anhydrase inhibitor/beta blocker fixed-combination eye drops; A, alpha agonist eye drops; AB, alpha agonist/beta blocker fixed-combination eye drops; B, beta blocker eye drops; PB, prostaglandin analog/beta blocker fixed-combination eye drops.
(TIFF)

S3 Fig Percentage of patients who received each type of glaucoma eye drop prescription. P, prostaglandin analog eye drops; CB, carbonic anhydrase inhibitor/beta blocker fixed-combination eye drops; A, alpha agonist eye drops; AB, alpha agonist/beta blocker fixed-combination eye drops; B, beta blocker eye drops; PB, prostaglandin analog/beta blocker fixed-combination eye drops.
(TIFF)

S4 Fig Number of patients prescribed each glaucoma eye drop. P, prostaglandin analog eye drops; CB, carbonic anhydrase inhibitor/beta blocker fixed-combination eye drops; A, alpha agonist eye drops; AB, alpha agonist/beta blocker fixed-combination eye drops; B, beta blocker eye drops; PB, prostaglandin analog/beta blocker fixed-combination eye drops; C, carbonic anhydrase inhibitor eye drops; AC, alpha agonist/carbonic anhydrase inhibitor fixed-combination eye drops; M, pilocarpine eye drops.
(TIFF)

S5 Fig Percentage of patients prescribed each glaucoma eye drop. P, prostaglandin analog eye drops; CB, carbonic anhydrase inhibitor/beta blocker fixed-combination eye drops; A, alpha agonist eye drops; AB, alpha agonist/beta blocker fixed-combination eye drops; B, beta blocker eye drops; PB, prostaglandin analog/beta blocker fixed-combination eye drops; C, carbonic anhydrase inhibitor eye drops; AC, alpha agonist/carbonic anhydrase inhibitor fixed-combination eye drops; M, pilocarpine eye drops.
(TIFF)

S6 Fig Number of patients prescribed each active ingredient of glaucoma eye drop. (TIFF)

S7 Fig Percentage of patients prescribed each active ingredient of glaucoma eye drop. (TIFF)

S8 Fig Percentage of patients who received each type of glaucoma eye drop prescription according to age group in 2020. CB, carbonic anhydrase inhibitor/beta blocker fixed-combination eye drops; A, alpha agonist eye drops; B, beta blocker eye drops; P, prostaglandin analog eye drops; AB, alpha agonist/beta blocker fixed-combination eye drops.
(TIFF)
