# Supplementary material for: Prescribing trends of glaucoma medication in Korea from 2007 to 2020: A nationwide population-based study
Source: PLoS One. 2024 Jul 11;19(7):e0305619. doi: 10.1371/journal.pone.0305619 (PMC11238952; doi:10.1371/journal.pone.0305619)
Supplement: S1 Table — (DOCX) [file pone.0305619.s001.docx]

S1 Table. Simple linear regression for analyzing the number of patients prescribed glaucoma eye drops according to age and sex

| Sex  Age group | Intercept | Regression coefficient | *P* value |
| --- | --- | --- | --- |
| Men |  |  |  |
| 0-9 | -23124.1 | 11.7 | <0.001 |
| 10-19 | -1677.4 | 2.0 | 0.918 |
| 20-29 | -454218.5 | 229.1 | <0.001 |
| 30-39 | -1111000.0 | 558.9 | <0.001 |
| 40-49 | -4015000.0 | 2008.0 | <0.001 |
| 50-59 | -8264000.0 | 4127.0 | <0.001 |
| 60-69 | -11400000.0 | 5693.0 | <0.001 |
| 70-79 | -11800000.0 | 5887.0 | <0.001 |
| 80-89 | -5048453.2 | 2515.9 | <0.001 |
| 90- | -354620.8 | 176.7 | <0.001 |
| Women |  |  |  |
| 0-9 | -51407.6 | 25.8 | <0.001 |
| 10-19 | 2271.8 | -0.3 | 0.984 |
| 20-29 | -262553.6 | 133.4 | 0.024 |
| 30-39 | -854699.2 | 428.9 | <0.001 |
| 40-49 | -2943000.0 | 1470.0 | <0.001 |
| 50-59 | -6238000.0 | 3115.0 | <0.001 |
| 60-69 | -9348877.9 | 4669.8 | <0.001 |
| 70-79 | -12240000.0 | 6110.0 | <0.001 |
| 80-89 | -7710767.6 | 3843.3 | <0.001 |
| 90- | -713577.9 | 355.5 | <0.001 |
| Total | -82837040.0 | 41362.0 | <0.001 |
