# Supplementary material for: Prescribing trends of glaucoma medication in Korea from 2007 to 2020: A nationwide population-based study
Source: PLoS One. 2024 Jul 11;19(7):e0305619. doi: 10.1371/journal.pone.0305619 (PMC11238952; doi:10.1371/journal.pone.0305619)
Supplement: S2 Table — (DOCX) [file pone.0305619.s002.docx]

S2 Table. Simple linear regression for analyzing the prevalence of patients (stratified according to age and sex) prescribed glaucoma eye drops per year

| Sex  Age group | Prevalence of patients who were prescribed glaucoma eye drops with a diagnosis of glaucoma (%) | | | | | | | | | | | | | | Intercept | Regression coefficient | *P* value |
| --- | --- | --- | --- | --- | --- | --- | --- | --- | --- | --- | --- | --- | --- | --- | --- | --- | --- |
|  | 2007 | 2008 | 2009 | 2010 | 2011 | 2012 | 2013 | 2014 | 2015 | 2016 | 2017 | 2018 | 2019 | 2020 |  |  |  |
| Men |  |  |  |  |  |  |  |  |  |  |  |  |  |  |  |  |  |
| 0-9 | 0.016 | 0.016 | 0.016 | 0.018 | 0.017 | 0.018 | 0.020 | 0.019 | 0.023 | 0.022 | 0.024 | 0.027 | 0.028 | 0.028 | -2.050 | 0.001 | <0.001 |
| 10-19 | 0.060 | 0.063 | 0.065 | 0.071 | 0.071 | 0.079 | 0.079 | 0.080 | 0.092 | 0.090 | 0.089 | 0.094 | 0.092 | 0.076 | -4.563 | 0.002 | <0.001 |
| 20-29 | 0.136 | 0.139 | 0.146 | 0.170 | 0.187 | 0.217 | 0.236 | 0.231 | 0.219 | 0.203 | 0.209 | 0.207 | 0.212 | 0.212 | -11.727 | 0.006 | 0.003 |
| 30-39 | 0.237 | 0.246 | 0.259 | 0.289 | 0.322 | 0.354 | 0.374 | 0.384 | 0.385 | 0.390 | 0.410 | 0.416 | 0.435 | 0.435 | -32.200 | 0.016 | <0.001 |
| 40-49 | 0.414 | 0.439 | 0.480 | 0.510 | 0.554 | 0.605 | 0.653 | 0.715 | 0.726 | 0.775 | 0.841 | 0.900 | 0.945 | 0.961 | -89.171 | 0.045 | <0.001 |
| 50-59 | 0.862 | 0.944 | 1.045 | 0.931 | 1.072 | 1.210 | 1.321 | 1.442 | 1.256 | 1.334 | 1.440 | 1.526 | 1.640 | 1.694 | -122.300 | 0.061 | <0.001 |
| 60-69 | 1.672 | 1.866 | 2.083 | 2.043 | 2.250 | 2.431 | 2.649 | 2.927 | 2.595 | 2.732 | 2.878 | 2.990 | 3.145 | 3.142 | -220.400 | 0.111 | <0.001 |
| 70-79 | 2.565 | 2.983 | 3.509 | 2.930 | 3.390 | 3.865 | 4.467 | 5.003 | 4.496 | 4.768 | 5.041 | 5.366 | 5.741 | 5.794 | -496.325 | 0.249 | <0.001 |
| 80-89 | 2.624 | 3.167 | 3.852 | 3.237 | 3.750 | 4.359 | 5.132 | 6.008 | 4.983 | 5.252 | 5.606 | 5.930 | 6.315 | 6.381 | -566.157 | 0.284 | <0.001 |
| 90- | 1.878 | 2.290 | 3.075 | 2.323 | 2.808 | 3.453 | 4.317 | 5.180 | 3.739 | 3.972 | 4.203 | 4.630 | 4.750 | 4.980 | -448.055 | 0.224 | <0.001 |
| Women |  |  |  |  |  |  |  |  |  |  |  |  |  |  |  |  |  |
| 0-9 | 0.013 | 0.011 | 0.013 | 0.015 | 0.013 | 0.017 | 0.019 | 0.020 | 0.028 | 0.026 | 0.029 | 0.025 | 0.027 | 0.030 | -3.086 | 0.002 | <0.001 |
| 10-19 | 0.047 | 0.048 | 0.047 | 0.052 | 0.054 | 0.060 | 0.063 | 0.064 | 0.071 | 0.067 | 0.066 | 0.061 | 0.063 | 0.061 | -2.906 | 0.001 | <0.001 |
| 20-29 | 0.128 | 0.130 | 0.143 | 0.170 | 0.189 | 0.213 | 0.243 | 0.229 | 0.217 | 0.197 | 0.198 | 0.193 | 0.190 | 0.191 | -9.384 | 0.005 | 0.033 |
| 30-39 | 0.143 | 0.148 | 0.156 | 0.181 | 0.205 | 0.232 | 0.248 | 0.253 | 0.269 | 0.275 | 0.293 | 0.299 | 0.316 | 0.314 | -29.000 | 0.015 | <0.001 |
| 40-49 | 0.233 | 0.251 | 0.267 | 0.280 | 0.311 | 0.346 | 0.379 | 0.422 | 0.446 | 0.487 | 0.543 | 0.597 | 0.643 | 0.665 | -70.217 | 0.035 | <0.001 |
| 50-59 | 0.622 | 0.675 | 0.737 | 0.646 | 0.753 | 0.847 | 0.929 | 1.024 | 0.915 | 0.990 | 1.062 | 1.128 | 1.222 | 1.272 | -98.131 | 0.049 | <0.001 |
| 60-69 | 1.480 | 1.617 | 1.789 | 1.771 | 1.945 | 2.076 | 2.232 | 2.417 | 2.222 | 2.352 | 2.447 | 2.522 | 2.653 | 2.611 | -175.200 | 0.088 | <0.001 |
| 70-79 | 2.278 | 2.609 | 2.998 | 2.680 | 3.053 | 3.442 | 3.898 | 4.296 | 4.141 | 4.434 | 4.638 | 4.879 | 5.218 | 5.180 | -467.276 | 0.234 | <0.001 |
| 80-89 | 1.854 | 2.255 | 2.703 | 2.297 | 2.710 | 3.145 | 3.670 | 4.251 | 3.734 | 4.084 | 4.412 | 4.733 | 5.089 | 5.167 | -512.125 | 0.256 | <0.001 |
| 90- | 1.063 | 1.241 | 1.502 | 1.165 | 1.386 | 1.672 | 2.210 | 2.546 | 1.881 | 2.071 | 2.217 | 2.479 | 2.824 | 2.816 | -266.888 | 0.134 | <0.001 |
| Total | 0.449 | 0.496 | 0.554 | 0.608 | 0.685 | 0.764 | 0.847 | 0.928 | 0.965 | 1.047 | 1.144 | 1.237 | 1.355 | 1.408 | -150.900 | 0.075 | <0.001 |
