# Supplementary material for: Prescribing trends of glaucoma medication in Korea from 2007 to 2020: A nationwide population-based study
Source: PLoS One. 2024 Jul 11;19(7):e0305619. doi: 10.1371/journal.pone.0305619 (PMC11238952; doi:10.1371/journal.pone.0305619)
Supplement: S3 Table — (DOCX) [file pone.0305619.s003.docx]

S3 Table. Simple linear regression for analyzing the number and percentage of patients who received each type of glaucoma eye drop prescription per year

|  | Number of patients | | | Percentage of patients | | |
| --- | --- | --- | --- | --- | --- | --- |
| Type | Intercept | Regression coefficient | *P* value | Intercept | Regression coefficient | *P* value |
| P | -20850000.0 | 10410.0 | <0.001 | 261.326 | -0.117 | <0.001 |
| CB | -24680000.0 | 12310.0 | <0.001 | -1790.234 | 0.900 | <0.001 |
| P+CB | -7570828.1 | 3776.3 | <0.001 | -394.652 | 0.200 | <0.001 |
| A | -6247895.0 | 3122.1 | <0.001 | 184.688 | -0.087 | 0.005 |
| AB | -7714275.5 | 3844.9 | <0.001 | -727.270 | 0.364 | <0.001 |
| B | -432891.1 | 232.3 | 0.120 | 1739.917 | -0.860 | <0.001 |
| P+CB+A | -4691000.0 | 2340.0 | <0.001 | -260.355 | 0.131 | <0.001 |
| CB+A | -3992000.0 | 1993.0 | <0.001 | -51.711 | 0.028 | 0.037 |
| PB | -1731312.5 | 865.0 | 0.001 | 124.061 | -0.060 | 0.165 |
| P+AB | -1729000.0 | 861.9 | <0.001 | -151.270 | 0.076 | <0.001 |
| P+A | -938606.0 | 470.1 | <0.001 | 129.753 | -0.064 | <0.001 |
| C | -211375.3 | 107.3 | 0.484 | 185.862 | -0.092 | 0.090 |
| AC | -731598.8 | 365.0 | 0.621 | -33.012 | 0.017 | 0.903 |
| P+B | 363499.3 | -178.0 | <0.001 | 378.260 | -0.187 | <0.001 |
| PB+AC | -1869858.6 | 927.8 | <0.001 | -237.700 | 0.118 | <0.001 |
| M | 63866.2 | -30.6 | 0.080 | 140.568 | -0.070 | <0.001 |
| P+C | -107440.4 | 54.3 | 0.104 | 64.847 | -0.032 | 0.010 |
| PB+A | -100655.5 | 50.6 | 0.008 | 41.422 | -0.020 | <0.001 |
| P+AC | -665846.0 | 330.8 | 0.061 | -79.356 | 0.039 | 0.194 |
| P+C+AB | -181900.0 | 90.8 | <0.001 | -7.857 | 0.004 | 0.367 |
| C+AB | -147900.0 | 73.9 | <0.001 | -0.364 | 0.000 | 0.949 |
| CB+AB | -155100.0 | 77.3 | <0.001 | -17.076 | 0.009 | <0.001 |
| PB+CB | -76247.2 | 38.1 | <0.001 | 3.959 | -0.002 | 0.243 |
| P+CB+AB | -129800.0 | 64.6 | <0.001 | -16.340 | 0.008 | <0.001 |
| PB+C | -20967.4 | 10.7 | 0.230 | 21.105 | -0.010 | <0.001 |
| Others | 472015.4 | -230.7 | 0.003 | 550.701 | -0.272 | <0.001 |
| Total | -82836947.0 | 41362.0 | <0.001 |  |  |  |
| P = prostaglandin analog eye drops, CB = carbonic anhydrase inhibitor/beta blocker fixed-combination eye drops, A = alpha agonist eye drops, AB = alpha agonist/beta blocker fixed-combination eye drops, B = beta blocker eye drops, PB = prostaglandin analog/beta blocker fixed-combination eye drops, C = carbonic anhydrase inhibitor eye drops, AC = alpha agonist/carbonic anhydrase inhibitor fixed-combination eye drops, M = pilocarpine eye drops | | | | | | |
