# Supplementary material for: Prescribing trends of glaucoma medication in Korea from 2007 to 2020: A nationwide population-based study
Source: PLoS One. 2024 Jul 11;19(7):e0305619. doi: 10.1371/journal.pone.0305619 (PMC11238952; doi:10.1371/journal.pone.0305619)
Supplement: S4 Table — (DOCX) [file pone.0305619.s004.docx]

S4 Table. Simple linear regression for analyzing the number and percentage of patients prescribed each glaucoma eye drop per year

|  | Number of patients | | | Percentage of patients | | |
| --- | --- | --- | --- | --- | --- | --- |
| Eye drops | Intercept | Regression coefficient | *P* value | Intercept | Regression coefficient | *P* value |
| P | -36150000.0 | 18050.0 | <0.001 | 105.584 | -0.030 | 0.171 |
| CB | -41320000.0 | 20610.0 | <0.001 | -2397.571 | 1.210 | <0.001 |
| A | -15746676.0 | 7866.0 | <0.001 | 335.060 | -0.156 | 0.001 |
| AB | -10150000.0 | 5062.0 | <0.001 | -912.443 | 0.457 | <0.001 |
| B | 421829.5 | -188.0 | 0.356 | 2454.343 | -1.213 | <0.001 |
| PB | -2569053.0 | 1283.0 | 0.001 | 134.624 | -0.065 | 0.282 |
| C | -447200.4 | 227.6 | 0.333 | 441.182 | -0.218 | 0.014 |
| AC | -3530012.2 | 1754.2 | 0.095 | -399.271 | 0.199 | 0.284 |
| M | 375820.8 | -184.3 | <0.001 | 362.240 | -0.179 | <0.001 |
| Total | -82836947.0 | 41362.0 | <0.001 | 190.411 | -0.030 | 0.167 |
| P = prostaglandin analog eye drops, CB = carbonic anhydrase inhibitor/beta blocker fixed-combination eye drops, A = alpha agonist eye drops, AB = alpha agonist/beta blocker fixed-combination eye drops, B = beta blocker eye drops, PB = prostaglandin analog/beta blocker fixed-combination eye drops, C = carbonic anhydrase inhibitor eye drops, AC = alpha agonist/carbonic anhydrase inhibitor fixed-combination eye drops, M = pilocarpine eye drops | | | | | | |
