# Supplementary material for: Prescribing trends of glaucoma medication in Korea from 2007 to 2020: A nationwide population-based study
Source: PLoS One. 2024 Jul 11;19(7):e0305619. doi: 10.1371/journal.pone.0305619 (PMC11238952; doi:10.1371/journal.pone.0305619)
Supplement: S5 Table — (DOCX) [file pone.0305619.s005.docx]

S5 Table. Simple linear regression for analyzing the number and percentage of patients prescribed each active ingredient of glaucoma eye drop per year

|  | Number of patients | | | Percentage of patients | | |
| --- | --- | --- | --- | --- | --- | --- |
| Active ingredients | Intercept | Regression coefficient | *P* value | Intercept | Regression coefficient | *P* value |
| Beta blocker | -53620000.0 | 26760.0 | <0.001 | -721.024 | 0.388 | <0.001 |
| Prostaglandin analog | -38720000.0 | 19330.0 | <0.001 | 240.256 | -0.096 | 0.056 |
| Carbonic anhydrase inhibitor | -43950000.0 | 21920.0 | <0.001 | -2288.998 | 1.157 | <0.001 |
| Alpha agonist | -28090000.0 | 14020.0 | <0.001 | -909.974 | 0.467 | <0.001 |
| Pilocarpine | 375820.8 | -184.3 | <0.001 | 362.240 | -0.179 | <0.001 |
| Total | -82836947.0 | 41362.0 | <0.001 | -3317.500 | 1.737 | <0.001 |
