# Supplementary material for: Prescribing trends of glaucoma medication in Korea from 2007 to 2020: A nationwide population-based study
Source: PLoS One. 2024 Jul 11;19(7):e0305619. doi: 10.1371/journal.pone.0305619 (PMC11238952; doi:10.1371/journal.pone.0305619)
Supplement: S6 Table — (DOCX) [file pone.0305619.s006.docx]

S6 Table. Number of patients (men) who received each type of glaucoma eye drop prescription according to age group in 2020

|  | Men | | | | | | | | | | | | | | | | | | | |
| --- | --- | --- | --- | --- | --- | --- | --- | --- | --- | --- | --- | --- | --- | --- | --- | --- | --- | --- | --- | --- |
| Drug | 0-9 years | | 10-19 years | | 20-29 years | | 30-39 years | | 40-49 years | | 50-59 years | | 60-69 years | | 70-79 years | | 80-89 years | | 90- years | |
| P | 31 | (5.4) | 199 | (10.7) | 1,002 | (12.7) | 2,706 | (16.8) | 8,529 | (21.2) | 16,394 | (22.5) | 24,890 | (24.5) | 25,159 | (26.6) | 10,299 | (27.5) | 703 | (27.2) |
| CB | 161 | (28.4) | 743 | (39.9) | 3,063 | (38.9) | 5,575 | (34.6) | 11,707 | (29.0) | 19,653 | (27.0) | 25,144 | (24.8) | 21,125 | (22.4) | 7,492 | (20.0) | 479 | (18.6) |
| P+CB | 73 | (12.9) | 98 | (5.3) | 441 | (5.6) | 1,131 | (7.0) | 3,448 | (8.6) | 6,326 | (8.7) | 9,034 | (8.9) | 8,653 | (9.2) | 3,609 | (9.6) | 231 | (8.9) |
| A | 137 | (24.2) | 207 | (11.1) | 746 | (9.5) | 1,214 | (7.5) | 2,482 | (6.2) | 4,955 | (6.8) | 6,865 | (6.8) | 6,674 | (7.1) | 2,621 | (7.0) | 185 | (7.2) |
| AB | 13 | (2.2) | 117 | (6.3) | 591 | (7.5) | 1,146 | (7.1) | 2,842 | (7.1) | 4,982 | (6.8) | 7,010 | (6.9) | 6,239 | (6.6) | 2,336 | (6.2) | 149 | (5.8) |
| B | 78 | (13.8) | 98 | (5.3) | 366 | (4.7) | 579 | (3.6) | 1,559 | (3.9) | 2,773 | (3.8) | 4,413 | (4.4) | 4,237 | (4.5) | 1,778 | (4.7) | 155 | (6.0) |
| P+CB+A | 27 | (4.8) | 108 | (5.8) | 426 | (5.4) | 903 | (5.6) | 2,391 | (5.9) | 4,621 | (6.3) | 6,139 | (6.1) | 5,956 | (6.3) | 2,415 | (6.4) | 170 | (6.6) |
| CB+A | 14 | (2.5) | 159 | (8.6) | 628 | (8.0) | 1,244 | (7.7) | 2,574 | (6.4) | 4,403 | (6.0) | 5,297 | (5.2) | 4,274 | (4.5) | 1,684 | (4.5) | 103 | (4.0) |
| PB | 4 | (0.7) | 13 | (0.7) | 123 | (1.6) | 412 | (2.6) | 1,236 | (3.1) | 2,213 | (3.0) | 3,238 | (3.2) | 2,850 | (3.0) | 1,194 | (3.2) | 88 | (3.4) |
| P+AB | 1 | (0.1) | 17 | (0.9) | 87 | (1.1) | 221 | (1.4) | 704 | (1.7) | 1,292 | (1.8) | 1,821 | (1.8) | 1,824 | (1.9) | 774 | (2.1) | 52 | (2.0) |
| P+A | 2 | (0.3) | 16 | (0.8) | 64 | (0.8) | 175 | (1.1) | 503 | (1.2) | 958 | (1.3) | 1,476 | (1.5) | 1,674 | (1.8) | 752 | (2.0) | 54 | (2.1) |
| C | 18 | (3.1) | 33 | (1.7) | 68 | (0.9) | 151 | (0.9) | 420 | (1.0) | 538 | (0.7) | 696 | (0.7) | 691 | (0.7) | 263 | (0.7) | 19 | (0.8) |
| AC | 0 | (0.0) | 4 | (0.2) | 34 | (0.4) | 99 | (0.6) | 207 | (0.5) | 415 | (0.6) | 614 | (0.6) | 656 | (0.7) | 265 | (0.7) | 24 | (0.9) |
| P+B | 1 | (0.1) | 2 | (0.1) | 14 | (0.2) | 46 | (0.3) | 155 | (0.4) | 372 | (0.5) | 596 | (0.6) | 608 | (0.6) | 330 | (0.9) | 29 | (1.1) |
| PB+AC | 0 | (0.1) | 8 | (0.4) | 46 | (0.6) | 116 | (0.7) | 312 | (0.8) | 582 | (0.8) | 782 | (0.8) | 686 | (0.7) | 275 | (0.7) | 27 | (1.1) |
| M | 1 | (0.2) | 9 | (0.5) | 32 | (0.4) | 54 | (0.3) | 135 | (0.3) | 312 | (0.4) | 361 | (0.4) | 187 | (0.2) | 54 | (0.1) | 5 | (0.2) |
| P+C | 4 | (0.6) | 2 | (0.1) | 6 | (0.1) | 26 | (0.2) | 95 | (0.2) | 193 | (0.3) | 299 | (0.3) | 353 | (0.4) | 188 | (0.5) | 20 | (0.8) |
| PB+A | 0 | (0.0) | 0 | (0.0) | 12 | (0.2) | 31 | (0.2) | 127 | (0.3) | 248 | (0.3) | 338 | (0.3) | 307 | (0.3) | 153 | (0.4) | 10 | (0.4) |
| P+AC | 0 | (0.0) | 1 | (0.1) | 11 | (0.1) | 34 | (0.2) | 102 | (0.3) | 192 | (0.3) | 300 | (0.3) | 365 | (0.4) | 142 | (0.4) | 16 | (0.6) |
| P+C+AB | 0 | (0.0) | 0 | (0.0) | 7 | (0.1) | 30 | (0.2) | 94 | (0.2) | 188 | (0.3) | 234 | (0.2) | 245 | (0.3) | 94 | (0.3) | 4 | (0.1) |
| C+AB | 0 | (0.0) | 7 | (0.4) | 16 | (0.2) | 48 | (0.3) | 89 | (0.2) | 152 | (0.2) | 197 | (0.2) | 157 | (0.2) | 59 | (0.2) | 2 | (0.1) |
| CB+AB | 0 | (0.0) | 4 | (0.2) | 16 | (0.2) | 21 | (0.1) | 59 | (0.1) | 126 | (0.2) | 162 | (0.2) | 132 | (0.1) | 44 | (0.1) | 3 | (0.1) |
| PB+CB | 0 | (0.0) | 0 | (0.0) | 7 | (0.1) | 13 | (0.1) | 63 | (0.2) | 112 | (0.2) | 156 | (0.2) | 127 | (0.1) | 62 | (0.2) | 6 | (0.2) |
| P+CB+AB | 0 | (0.0) | 1 | (0.1) | 8 | (0.1) | 22 | (0.1) | 68 | (0.2) | 98 | (0.1) | 143 | (0.1) | 134 | (0.1) | 52 | (0.1) | 4 | (0.1) |
| PB+C | 0 | (0.0) | 0 | (0.0) | 4 | (0.0) | 16 | (0.1) | 46 | (0.1) | 91 | (0.1) | 107 | (0.1) | 107 | (0.1) | 47 | (0.1) | 0 | (0.0) |
| Others | 3 | (0.5) | 15 | (0.8) | 56 | (0.7) | 121 | (0.8) | 351 | (0.9) | 721 | (1.0) | 1,105 | (1.1) | 1,072 | (1.1) | 511 | (1.4) | 42 | (1.6) |
| Total | 567 | (100.0) | 1,861 | (100.0) | 7,872 | (100.0) | 16,137 | (100.0) | 40,301 | (100.0) | 72,909 | (100.0) | 101,420 | (100.0) | 94,492 | (100.0) | 37,498 | (100.0) | 2,582 | (100.0) |
| P = prostaglandin analog eye drops, CB = carbonic anhydrase inhibitor/beta blocker fixed-combination eye drops, A = alpha agonist eye drops, AB = alpha agonist/beta blocker fixed-combination eye drops, B = beta blocker eye drops, PB = prostaglandin analog/beta blocker fixed-combination eye drops, C = carbonic anhydrase inhibitor eye drops, AC = alpha agonist/carbonic anhydrase inhibitor fixed-combination eye drops, M = pilocarpine eye drops | | | | | | | | | | | | | | | | | | | | |
