# Supplementary material for: Prescribing trends of glaucoma medication in Korea from 2007 to 2020: A nationwide population-based study
Source: PLoS One. 2024 Jul 11;19(7):e0305619. doi: 10.1371/journal.pone.0305619 (PMC11238952; doi:10.1371/journal.pone.0305619)
Supplement: S7 Table — (DOCX) [file pone.0305619.s007.docx]

S7 Table. Number of patients (women) who received each type of glaucoma eye drop prescription according to age group in 2020

|  | Women | | | | | | | | | | | | | | | | | | | |
| --- | --- | --- | --- | --- | --- | --- | --- | --- | --- | --- | --- | --- | --- | --- | --- | --- | --- | --- | --- | --- |
| Drug | 0-9 years | | 10-19 years | | 20-29 years | | 30-39 years | | 40-49 years | | 50-59 years | | 60-69 years | | 70-79 years | | 80-89 years | | 90- years | |
| P | 35 | (6.0) | 163 | (11.7) | 691 | (10.9) | 1,767 | (16.6) | 6,137 | (22.9) | 13,686 | (25.2) | 24,598 | (27.8) | 30,834 | (29.8) | 18,079 | (31.9) | 1,697 | (32.1) |
| CB | 142 | (24.2) | 544 | (39.0) | 2,580 | (40.8) | 3,723 | (35.0) | 8,001 | (29.8) | 15,367 | (28.2) | 22,130 | (25.0) | 22,891 | (22.1) | 10,728 | (18.9) | 876 | (16.6) |
| P+CB | 48 | (8.2) | 74 | (5.3) | 258 | (4.1) | 572 | (5.4) | 1,799 | (6.7) | 3,649 | (6.7) | 6,399 | (7.2) | 8,062 | (7.8) | 4,880 | (8.6) | 447 | (8.4) |
| A | 151 | (25.7) | 162 | (11.6) | 796 | (12.6) | 1,156 | (10.9) | 2,208 | (8.2) | 4,791 | (8.8) | 6,920 | (7.8) | 7,621 | (7.4) | 4,045 | (7.1) | 410 | (7.8) |
| AB | 16 | (2.8) | 105 | (7.5) | 536 | (8.5) | 877 | (8.2) | 2,137 | (8.0) | 4,118 | (7.6) | 6,868 | (7.8) | 7,583 | (7.3) | 3,727 | (6.6) | 281 | (5.3) |
| B | 151 | (25.7) | 81 | (5.8) | 373 | (5.9) | 586 | (5.5) | 1,572 | (5.9) | 3,307 | (6.1) | 5,954 | (6.7) | 6,816 | (6.6) | 3,428 | (6.0) | 352 | (6.7) |
| P+CB+A | 15 | (2.6) | 77 | (5.5) | 245 | (3.9) | 388 | (3.6) | 974 | (3.6) | 1,859 | (3.4) | 2,889 | (3.3) | 3,862 | (3.7) | 2,483 | (4.4) | 281 | (5.3) |
| CB+A | 5 | (0.8) | 79 | (5.7) | 408 | (6.4) | 701 | (6.6) | 1,253 | (4.7) | 2,123 | (3.9) | 3,154 | (3.6) | 3,507 | (3.4) | 1,923 | (3.4) | 214 | (4.0) |
| PB | 8 | (1.3) | 11 | (0.8) | 75 | (1.2) | 193 | (1.8) | 714 | (2.7) | 1,412 | (2.6) | 2,573 | (2.9) | 3,173 | (3.1) | 1,868 | (3.3) | 156 | (3.0) |
| P+AB | 0 | (0.0) | 19 | (1.3) | 47 | (0.7) | 124 | (1.2) | 352 | (1.3) | 679 | (1.2) | 1,254 | (1.4) | 1,702 | (1.6) | 971 | (1.7) | 94 | (1.8) |
| P+A | 1 | (0.1) | 20 | (1.5) | 40 | (0.6) | 88 | (0.8) | 295 | (1.1) | 555 | (1.0) | 993 | (1.1) | 1,482 | (1.4) | 1,066 | (1.9) | 113 | (2.1) |
| C | 8 | (1.3) | 18 | (1.3) | 67 | (1.1) | 82 | (0.8) | 274 | (1.0) | 517 | (0.9) | 819 | (0.9) | 934 | (0.9) | 452 | (0.8) | 47 | (0.9) |
| AC | 0 | (0.0) | 5 | (0.4) | 27 | (0.4) | 64 | (0.6) | 157 | (0.6) | 287 | (0.5) | 545 | (0.6) | 697 | (0.7) | 373 | (0.7) | 29 | (0.6) |
| P+B | 0 | (0.0) | 3 | (0.2) | 15 | (0.2) | 20 | (0.2) | 120 | (0.4) | 271 | (0.5) | 530 | (0.6) | 728 | (0.7) | 568 | (1.0) | 64 | (1.2) |
| PB+AC | 0 | (0.0) | 7 | (0.5) | 31 | (0.5) | 47 | (0.4) | 127 | (0.5) | 216 | (0.4) | 360 | (0.4) | 451 | (0.4) | 290 | (0.5) | 41 | (0.8) |
| M | 1 | (0.2) | 9 | (0.6) | 42 | (0.7) | 50 | (0.5) | 153 | (0.6) | 320 | (0.6) | 362 | (0.4) | 286 | (0.3) | 102 | (0.2) | 9 | (0.2) |
| P+C | 3 | (0.6) | 1 | (0.1) | 7 | (0.1) | 21 | (0.2) | 78 | (0.3) | 170 | (0.3) | 321 | (0.4) | 407 | (0.4) | 247 | (0.4) | 32 | (0.6) |
| PB+A | 0 | (0.0) | 0 | (0.0) | 5 | (0.1) | 24 | (0.2) | 56 | (0.2) | 110 | (0.2) | 184 | (0.2) | 261 | (0.3) | 167 | (0.3) | 13 | (0.3) |
| P+AC | 0 | (0.0) | 3 | (0.2) | 9 | (0.1) | 10 | (0.1) | 51 | (0.2) | 90 | (0.2) | 182 | (0.2) | 285 | (0.3) | 177 | (0.3) | 18 | (0.3) |
| P+C+AB | 0 | (0.1) | 0 | (0.0) | 7 | (0.1) | 13 | (0.1) | 37 | (0.1) | 78 | (0.1) | 133 | (0.1) | 171 | (0.2) | 110 | (0.2) | 9 | (0.2) |
| C+AB | 0 | (0.0) | 2 | (0.2) | 10 | (0.2) | 19 | (0.2) | 56 | (0.2) | 110 | (0.2) | 168 | (0.2) | 170 | (0.2) | 83 | (0.1) | 2 | (0.0) |
| CB+AB | 1 | (0.1) | 4 | (0.3) | 12 | (0.2) | 15 | (0.1) | 34 | (0.1) | 63 | (0.1) | 103 | (0.1) | 125 | (0.1) | 61 | (0.1) | 8 | (0.2) |
| PB+CB | 1 | (0.2) | 0 | (0.0) | 4 | (0.1) | 6 | (0.1) | 21 | (0.1) | 58 | (0.1) | 83 | (0.1) | 135 | (0.1) | 87 | (0.2) | 3 | (0.1) |
| P+CB+AB | 0 | (0.0) | 0 | (0.0) | 5 | (0.1) | 7 | (0.1) | 16 | (0.1) | 49 | (0.1) | 74 | (0.1) | 96 | (0.1) | 63 | (0.1) | 8 | (0.1) |
| PB+C | 0 | (0.0) | 0 | (0.0) | 2 | (0.0) | 14 | (0.1) | 21 | (0.1) | 48 | (0.1) | 106 | (0.1) | 117 | (0.1) | 78 | (0.1) | 4 | (0.1) |
| Others | 2 | (0.3) | 8 | (0.6) | 32 | (0.5) | 75 | (0.7) | 209 | (0.8) | 488 | (0.9) | 853 | (1.0) | 1,085 | (1.0) | 626 | (1.1) | 82 | (1.6) |
| Total | 589 | (100.0) | 1,395 | (100.0) | 6,325 | (100.0) | 10,643 | (100.0) | 26,851 | (100.0) | 54,419 | (100.0) | 88,555 | (100.0) | 103,483 | (100.0) | 56,680 | (100.0) | 5,292 | (100.0) |
| P = prostaglandin analog eye drops, CB = carbonic anhydrase inhibitor/beta blocker fixed-combination eye drops, A = alpha agonist eye drops, AB = alpha agonist/beta blocker fixed-combination eye drops, B = beta blocker eye drops, PB = prostaglandin analog/beta blocker fixed-combination eye drops, C = carbonic anhydrase inhibitor eye drops, AC = alpha agonist/carbonic anhydrase inhibitor fixed-combination eye drops, M = pilocarpine eye drops | | | | | | | | | | | | | | | | | | | | |
