# Supplementary material for: Prescribing trends of glaucoma medication in Korea from 2007 to 2020: A nationwide population-based study
Source: PLoS One. 2024 Jul 11;19(7):e0305619. doi: 10.1371/journal.pone.0305619 (PMC11238952; doi:10.1371/journal.pone.0305619)
Supplement: S8 Table — (DOCX) [file pone.0305619.s008.docx]

S8 Table. Simple linear regression for analyzing the percentage of patients who received each type of glaucoma eye drop prescription according to age group in 2020

|  | Percentage of patients | | |
| --- | --- | --- | --- |
| Type | Intercept | Regression coefficient | *P* value |
| P | 7.833 | 2.834 | < 0.001 |
| CB | 37.371 | -2.067 | 0.006 |
| P+CB | 6.806 | 0.196 | 0.326 |
| A | 15.682 | -1.267 | 0.025 |
| AB | 6.062 | 0.110 | 0.558 |
| B | 9.860 | -0.695 | 0.187 |
| P+CB+A | 4.540 | 0.095 | 0.128 |
| CB+A | 5.842 | -0.174 | 0.428 |
| PB | 1.087 | 0.284 | < 0.001 |
| P+AB | 0.637 | 0.161 | < 0.001 |
| P+A | 0.452 | 0.173 | < 0.001 |
| C | 1.568 | -0.112 | 0.012 |
| AC | 0.228 | 0.062 | 0.002 |
| P+B | -0.003 | 0.113 | < 0.001 |
| PB+AC | 0.312 | 0.054 | 0.014 |
| M | 0.462 | -0.024 | 0.153 |
| P+C | 0.198 | 0.031 | 0.169 |
| PB+A | 0.042 | 0.037 | < 0.001 |
| P+AC | 0.041 | 0.040 | < 0.001 |
| P+C+AB | 0.054 | 0.021 | 0.004 |
| C+AB | 0.189 | -0.004 | 0.679 |
| CB+AB | 0.168 | -0.005 | 0.368 |
| PB+CB | 0.052 | 0.011 | 0.016 |
| P+CB+AB | 0.041 | 0.012 | 0.003 |
| PB+C | 0.027 | 0.011 | 0.023 |
| Others | 0.450 | 0.104 | < 0.001 |
| P = prostaglandin analog eye drops, CB = carbonic anhydrase inhibitor/beta blocker fixed-combination eye drops, A = alpha agonist eye drops, AB = alpha agonist/beta blocker fixed-combination eye drops, B = beta blocker eye drops, PB = prostaglandin analog/beta blocker fixed-combination eye drops, C = carbonic anhydrase inhibitor eye drops, AC = alpha agonist/carbonic anhydrase inhibitor fixed-combination eye drops, M = pilocarpine eye drops | | | |
