# Supplementary figures and images for: Prescribing trends of glaucoma medication in Korea from 2007 to 2020: A nationwide population-based study
Source: PLoS One. 2024 Jul 11;19(7):e0305619. doi: 10.1371/journal.pone.0305619 (PMC11238952; doi:10.1371/journal.pone.0305619)

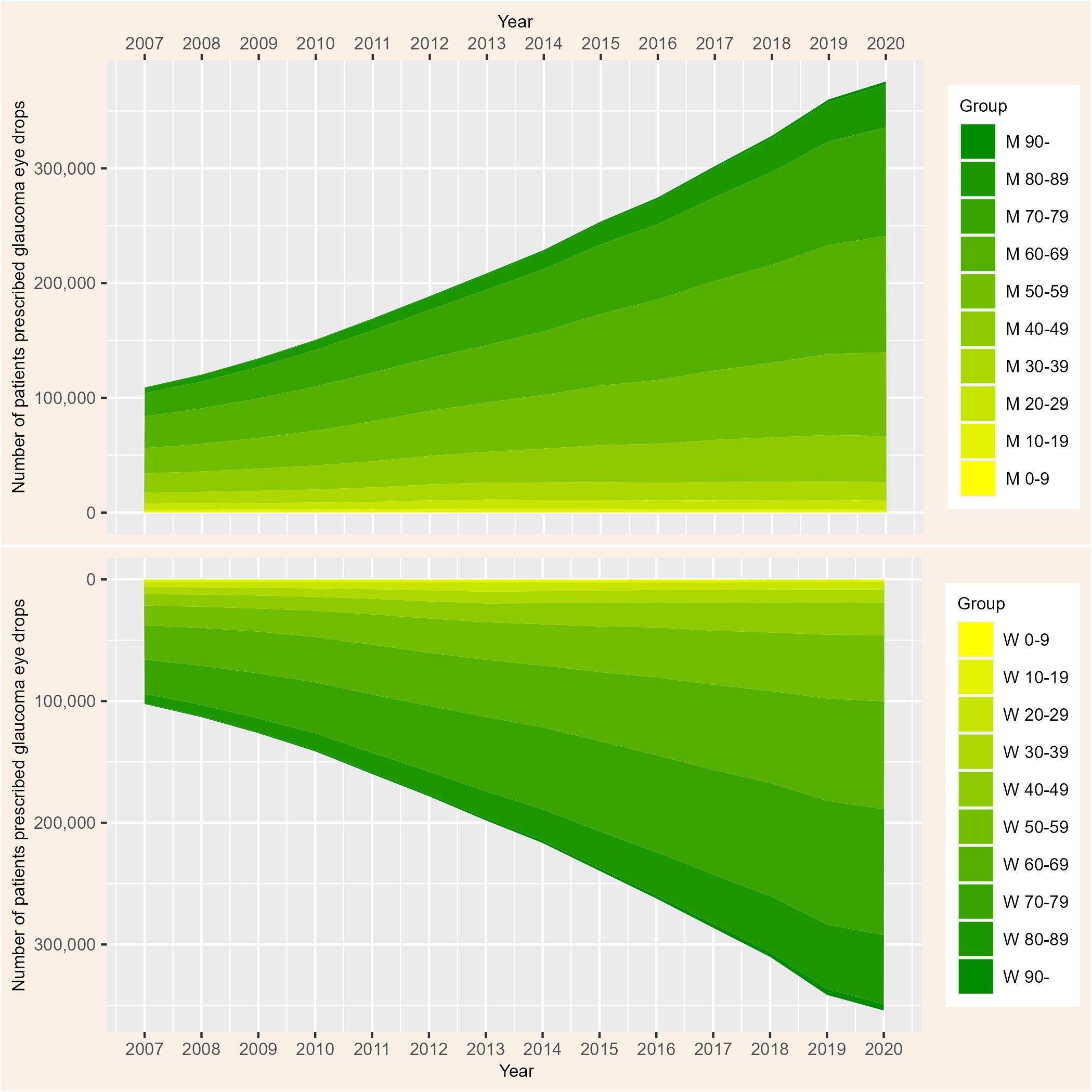

Supplement: S1 Fig — (TIFF) [file pone.0305619.s009.tiff]

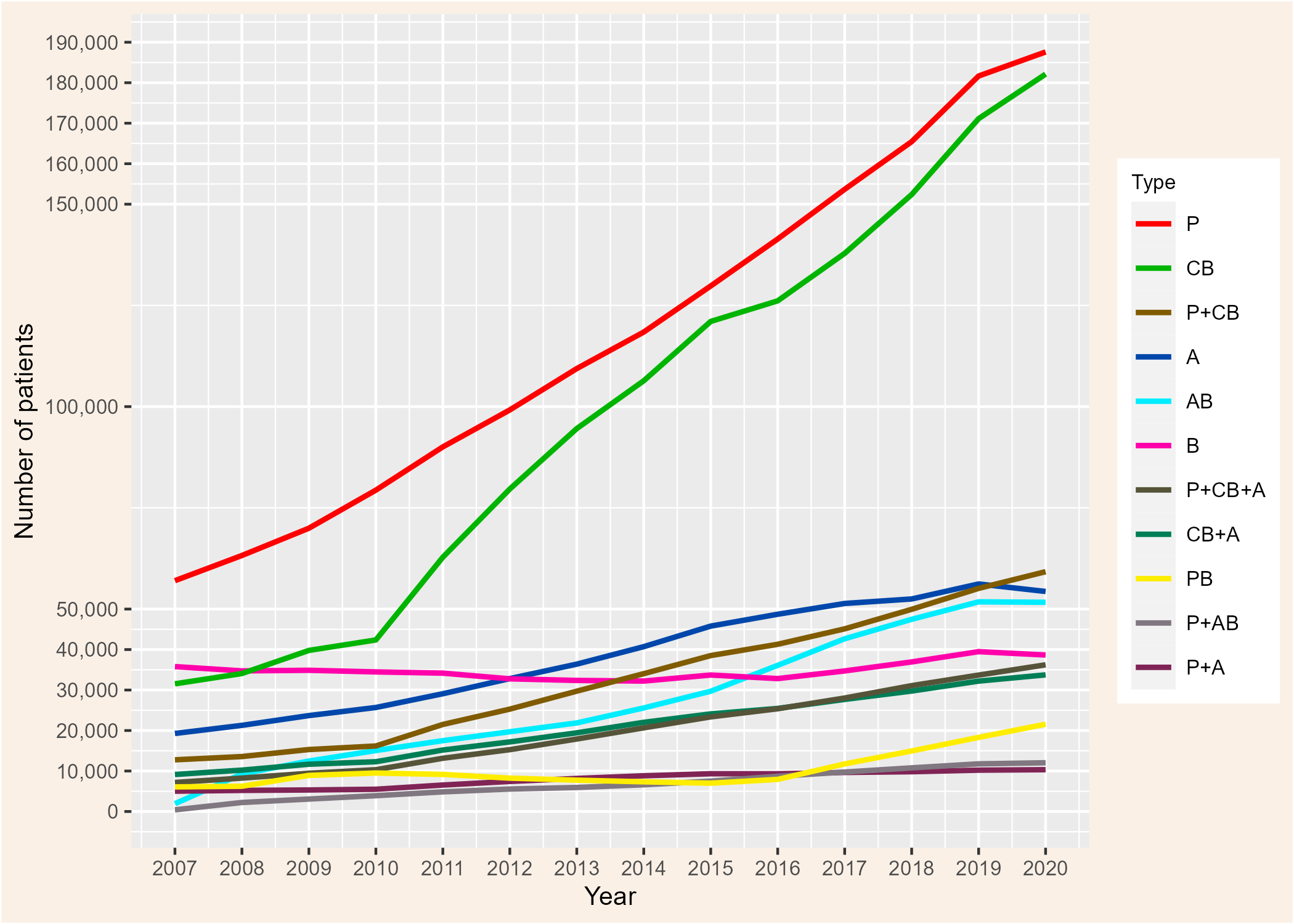

Supplement: S2 Fig — P, prostaglandin analog eye drops; CB, carbonic anhydrase inhibitor/beta blocker fixed-combination eye drops; A, alpha agonist eye drops; AB, alpha agonist/beta blocker fixed-combination eye drops; B, beta blocker eye drops; PB, prostaglandin analog/beta blocker fixed-combination eye drops. (TIFF) [file pone.0305619.s010.tiff]

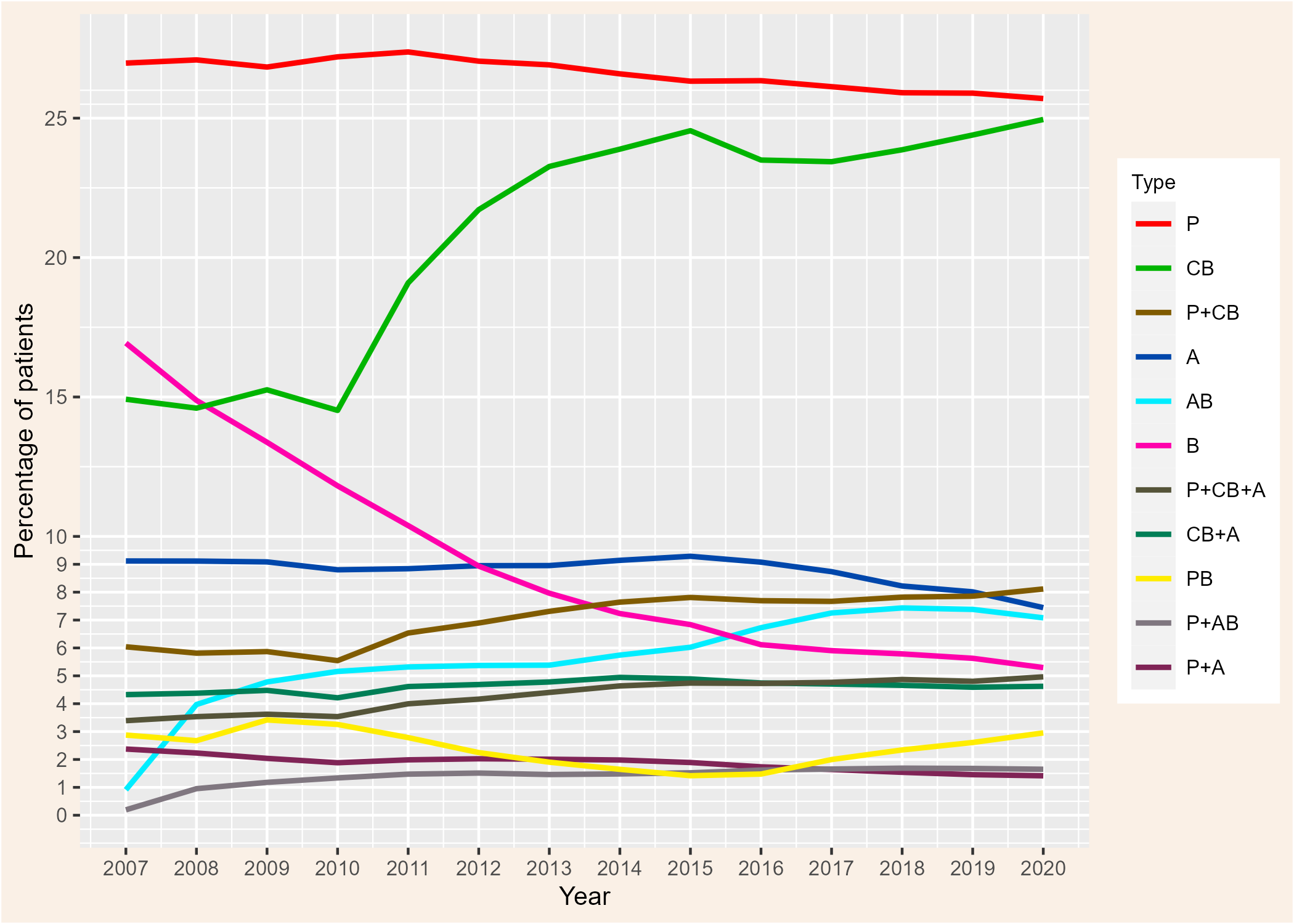

Supplement: S3 Fig — P, prostaglandin analog eye drops; CB, carbonic anhydrase inhibitor/beta blocker fixed-combination eye drops; A, alpha agonist eye drops; AB, alpha agonist/beta blocker fixed-combination eye drops; B, beta blocker eye drops; PB, prostaglandin analog/beta blocker fixed-combination eye drops. (TIFF) [file pone.0305619.s011.tiff]

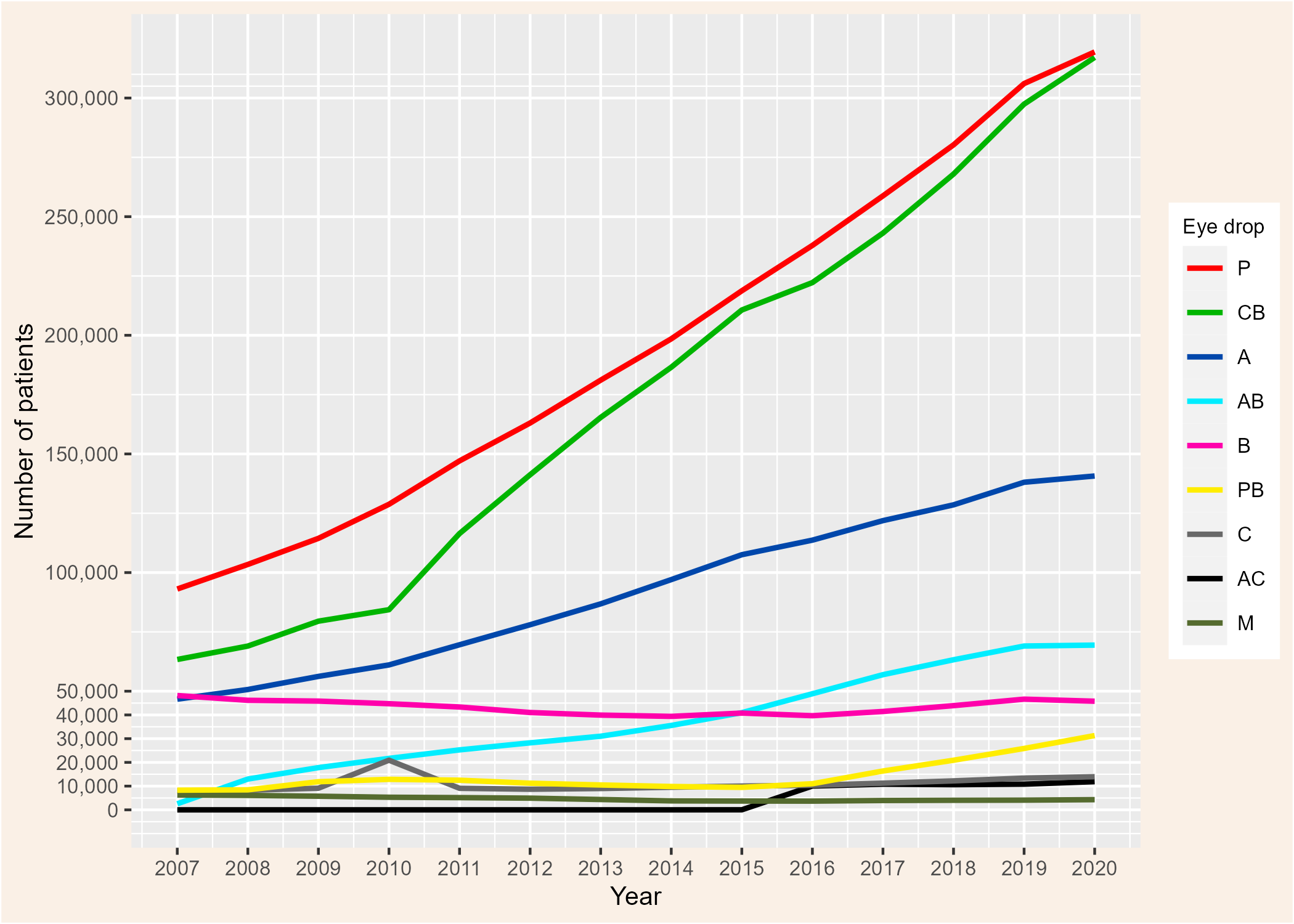

Supplement: S4 Fig — P, prostaglandin analog eye drops; CB, carbonic anhydrase inhibitor/beta blocker fixed-combination eye drops; A, alpha agonist eye drops; AB, alpha agonist/beta blocker fixed-combination eye drops; B, beta blocker eye drops; PB, prostaglandin analog/beta blocker fixed-combination eye drops; C, carbonic anhydrase inhibitor eye drops; AC, alpha agonist/carbonic anhydrase inhibitor fixed-combination eye drops; M, pilocarpine eye drops. (TIFF) [file pone.0305619.s012.tiff]

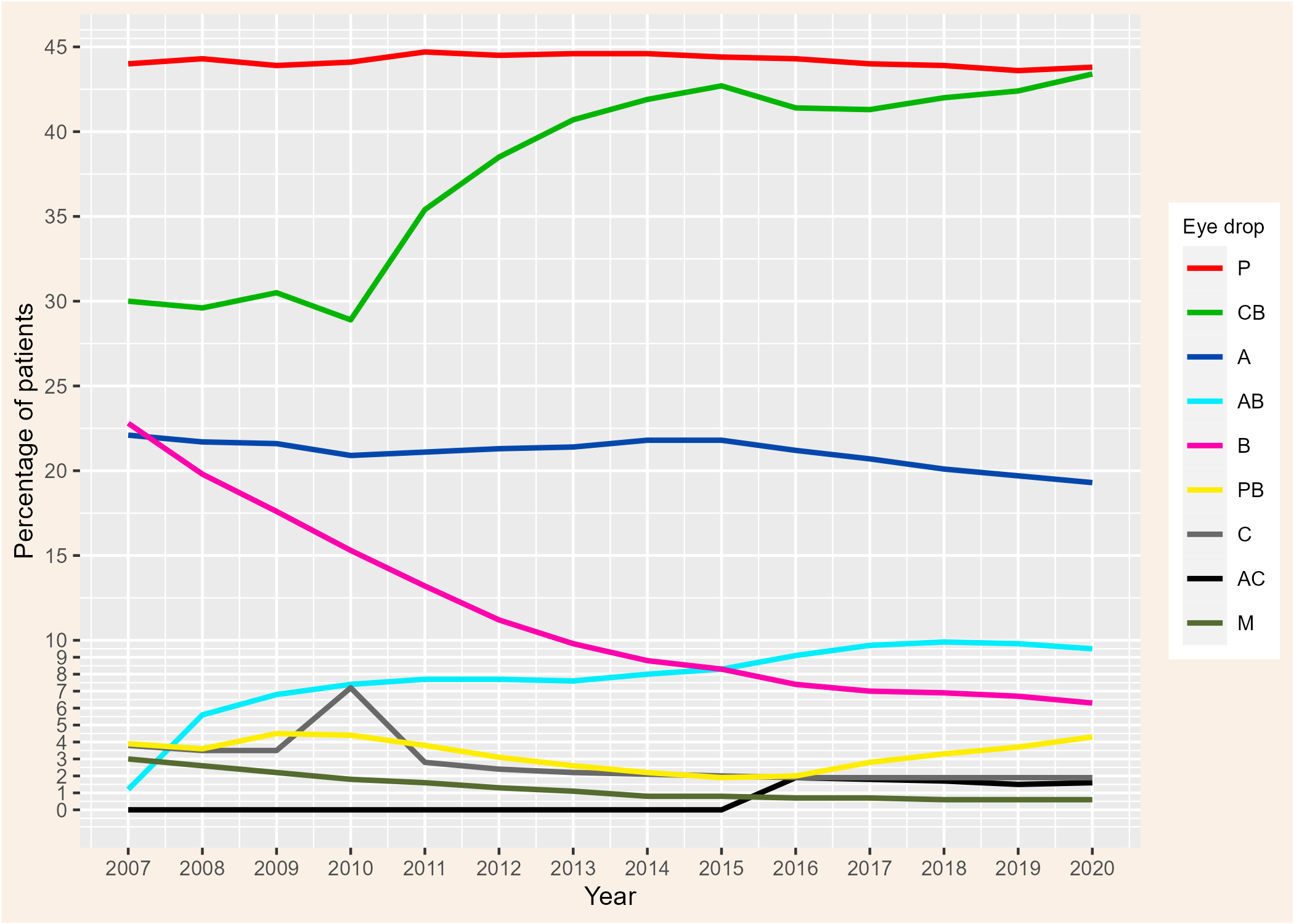

Supplement: S5 Fig — P, prostaglandin analog eye drops; CB, carbonic anhydrase inhibitor/beta blocker fixed-combination eye drops; A, alpha agonist eye drops; AB, alpha agonist/beta blocker fixed-combination eye drops; B, beta blocker eye drops; PB, prostaglandin analog/beta blocker fixed-combination eye drops; C, carbonic anhydrase inhibitor eye drops; AC, alpha agonist/carbonic anhydrase inhibitor fixed-combination eye drops; M, pilocarpine eye drops. (TIFF) [file pone.0305619.s013.tiff]

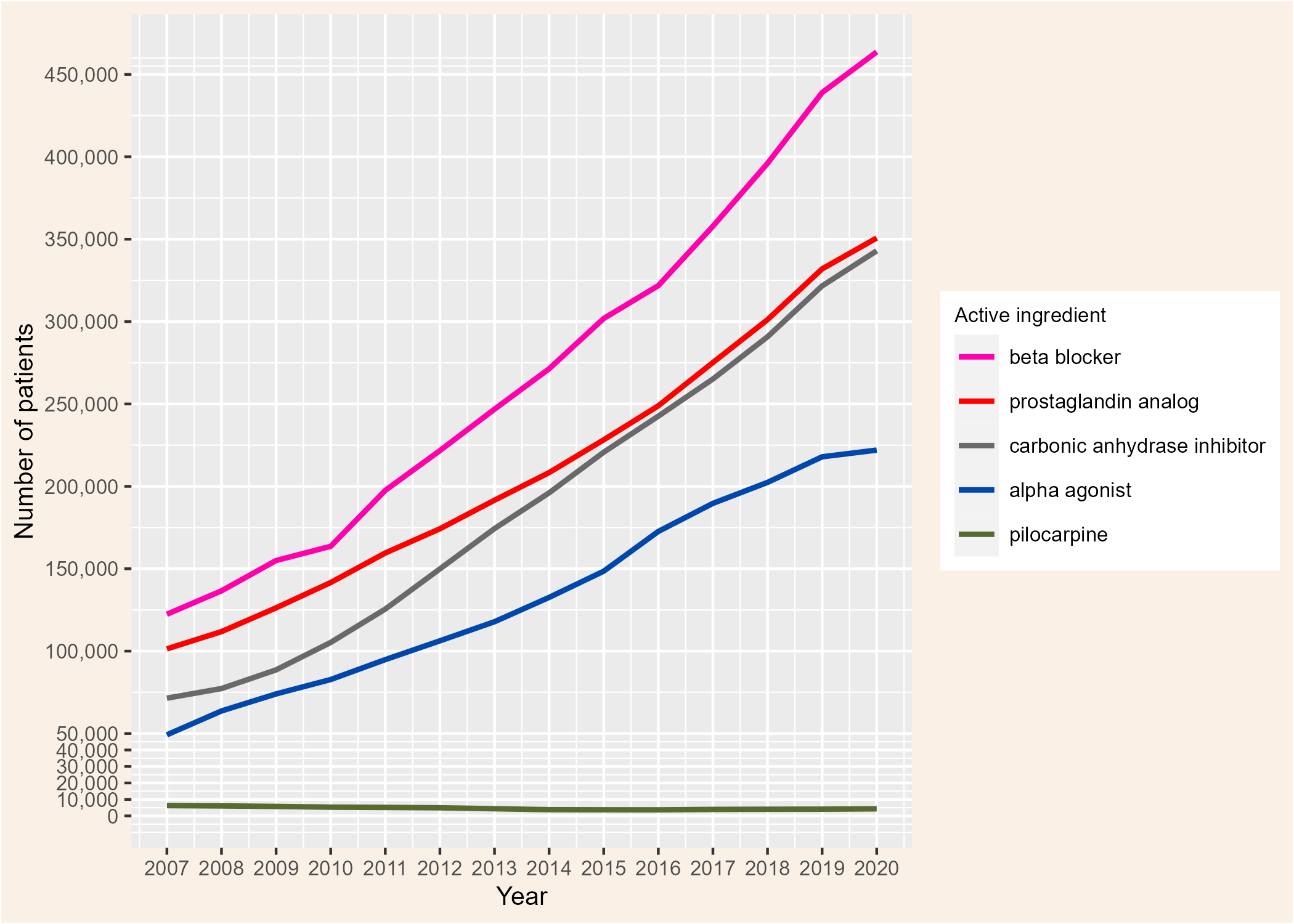

Supplement: S6 Fig — (TIFF) [file pone.0305619.s014.tiff]

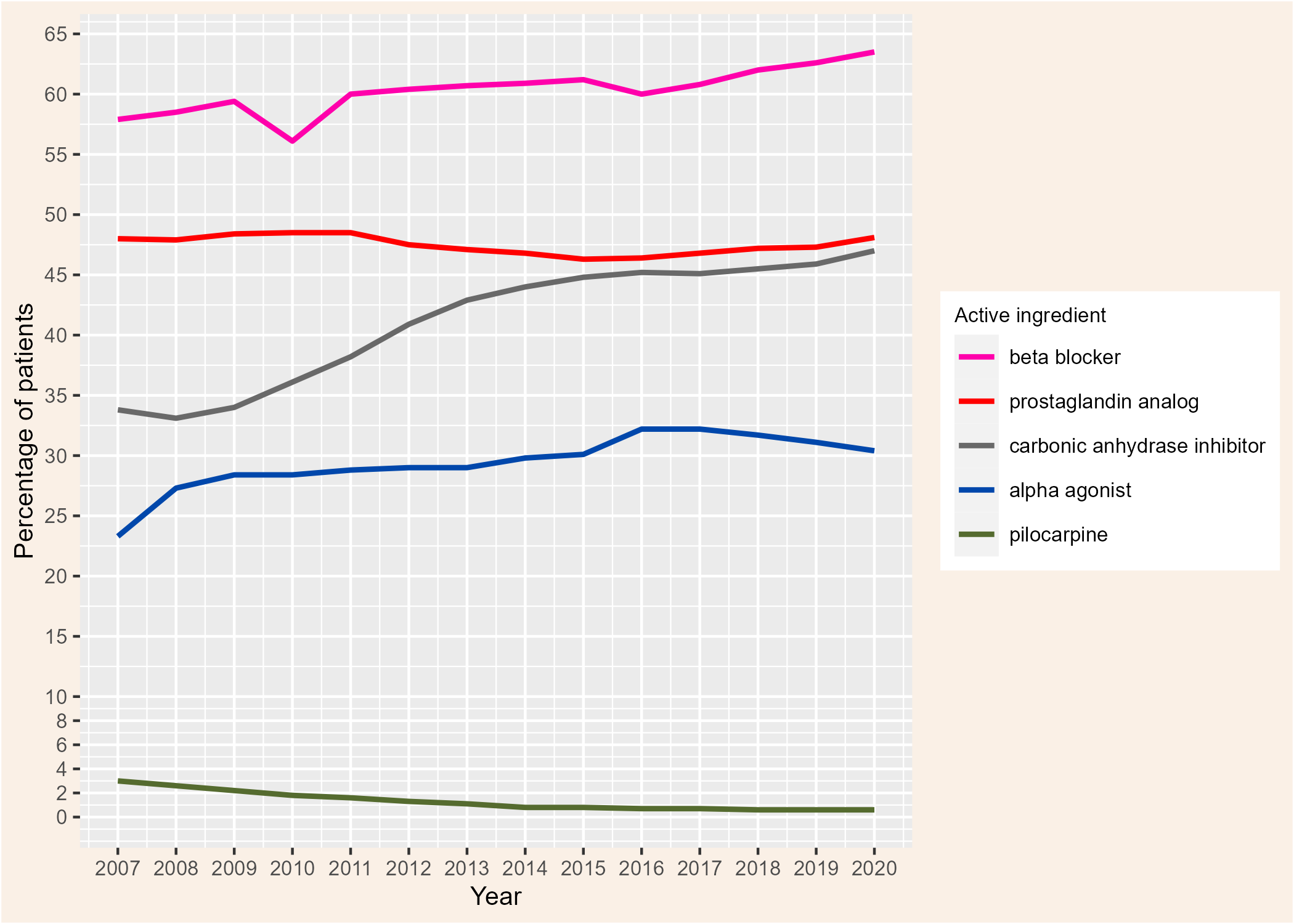

Supplement: S7 Fig — (TIFF) [file pone.0305619.s015.tiff]

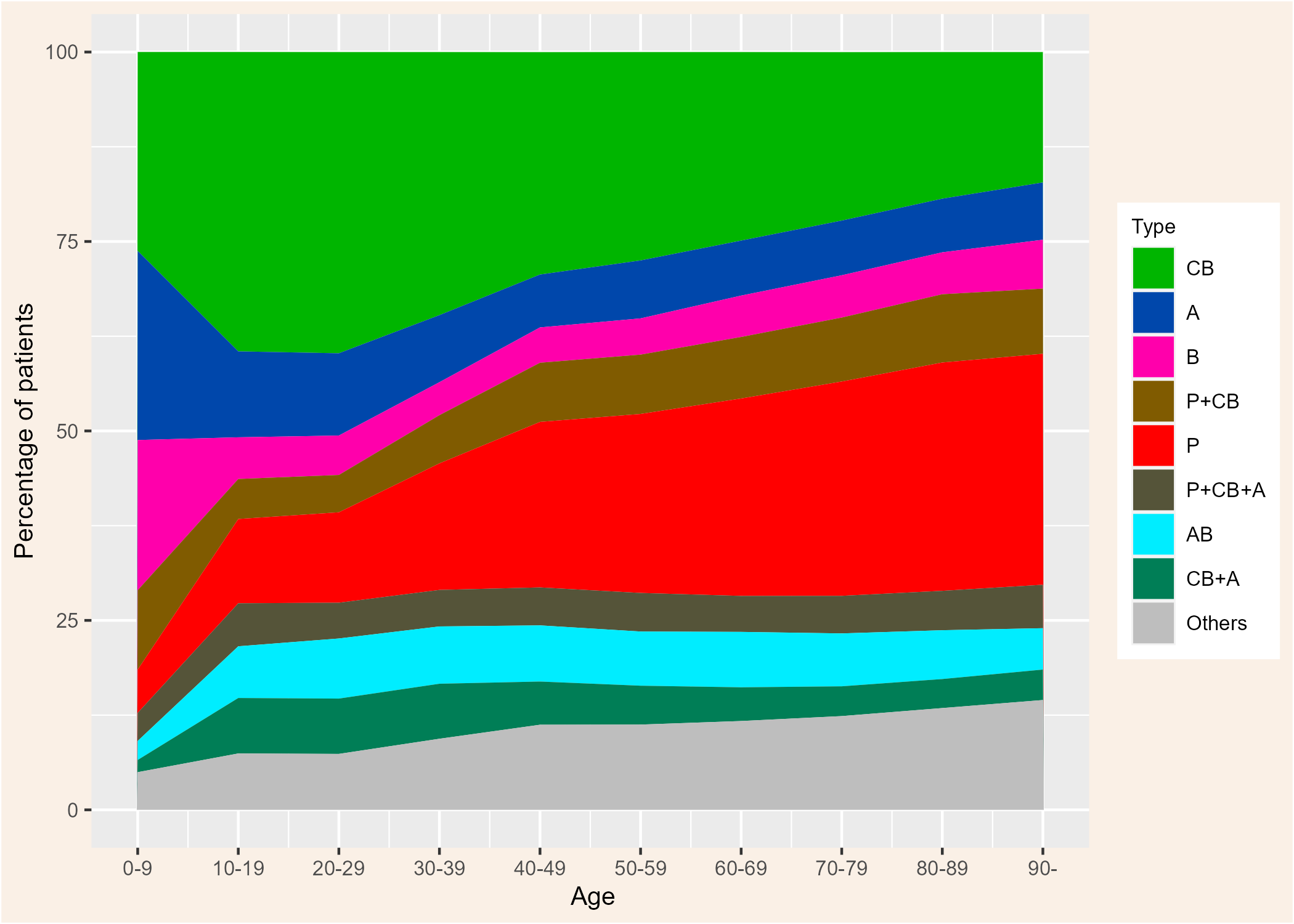

Supplement: S8 Fig — CB, carbonic anhydrase inhibitor/beta blocker fixed-combination eye drops; A, alpha agonist eye drops; B, beta blocker eye drops; P, prostaglandin analog eye drops; AB, alpha agonist/beta blocker fixed-combination eye drops. (TIFF) [file pone.0305619.s016.tiff]
